# The effect of a change in co-payment on prescription drug demand in a National Health System: The case of 15 drug families by price elasticity of demand

**DOI:** 10.1371/journal.pone.0213403

**Published:** 2019-03-27

**Authors:** Cristina Hernández-Izquierdo, Beatriz González López-Valcárcel, Stephen Morris, Mariya Melnychuk, Ignacio Abásolo Alessón

**Affiliations:** 1 Departamento de Economía Aplicada y Métodos Cuantitativos, Facultad de Economía, Empresa y Turismo, Universidad de La Laguna (ULL), San Cristóbal de La Laguna, Spain; 2 Departamento de Métodos Cuantitativos en Economía y Gestión, Facultad de Economía, Empresa y Turismo, Universidad de Las Palmas de Gran Canaria (ULPGC), Las Palmas de Gran Canaria, Spain; 3 Department of Applied Health Research, University College London (UCL), London, United Kingdom; 4 Departmento de Economía de la Empresa (ADO), Economía Aplicada II y Fundamentos de Análisis Económico, Universidad Rey Juan Carlos (URJC), Madrid, Spain; 5 Instituto Universitario de Desarrollo Regional, Facultad de Economía, Empresa y Turismo, Universidad de La Laguna (ULL), San Cristóbal de La Laguna, Spain; Universidad de Cantabria, SPAIN

## Abstract

**Objectives:**

To test the heterogeneity of the effect of a change in pharmaceutical cost-sharing by therapeutic groups in a Spanish region.

**Methods:**

Data: random sample (provided by the Canary Islands Health Service) of 40,471 people covered by the Spanish National Health System (SNHS) in the Canary Islands. The database includes individualised monthly-dispensed medications (prescribed by the SNHS) from one year before (August 2011) to one year after (June 2013) the Royal Decree Law 16/2012 (RDL 16/2012). Sample: two intervention groups (low-income pensioners and middle-income working population) and one control group (low-income working population). Empirical model: quasi-experimental difference-in-differences design to study the change in consumption (measured in number of monthly Defined Daily Dose (DDDs) per individual) among 13 therapeutic groups. The policy break indicator (three-level categorical variable) tested the existence of stockpiling between the reform’s announcement and its implementation. We ran 16 linear regression models (general, by therapeutic groups and by comorbidities) that considered whether the exclusion of some drugs from public provision impacted on consumption more than the co-payment increase.

**Results:**

General: Reduction (-13.04) in consumption after the reform’s implementation, which was fully compensated by a previous increase (16.60 i.e., stockpiling) among low-income pensioners. The middle-income working population maintained its trend of increasing consumption. Therapeutic groups: Reductions in consumption after the reform’s implementation among low-income pensioners in 7 of the 13 groups, which were fully compensated for by a previous increase (i.e., stockpiling) in 4 groups and partially compensated for in the remaining 3. The analysis without the excluded medicines provided fewer negative coefficients. Comorbidities: Reduction in consumption that was only slightly compensated for by a previous increase (i.e., stockpiling).

**Conclusions:**

The negative impact of cost-sharing produced, among low-income pensioners, a risk of loss of adherence to treatments, which could deteriorate the health status of individuals, especially among pensioners within the most inelastic therapeutic groups (associated with chronic diseases) and patients with comorbidities (also, associated with chronic diseases). Notwithstanding the above, this risk was more related to the exclusion of some drugs from provision than to the cost-sharing increase.

## 1. Introduction

The nature of the disease to be treated (e.g., chronic versus acute or severe versus mild) is a key factor to understand the behaviour of individuals’ pharmaceutical consumption [1]. Research has shown that patients with chronic diseases are less sensitive to drug price changes than non-chronic ones [2]. While it is true that most studies establish a general inelastic price-elasticity of drugs of around -0.2 [2–4], there is also evidence that the most inelastic drugs are those indicated for the treatment of chronic diseases with price-elasticity of approximately -0.08 [4].

Consequently, pharmaceutical co-payments have different impacts depending on the disease being treated, as well as many other determining factors (e.g., age, sex, income, social status, and educational level). A recent study using 15 different therapeutic drug groups, following ATC code rules (drugs grouped according to their Anatomical, Therapeutic and Chemical characteristics), analysed the sensitivity of each of these groups to a cost-sharing change and found that there were different price-elasticities depending on the therapeutic group [5]. In this regard, it is important to consider the heterogeneity of the effect of a pharmaceutical co-payment by therapeutic groups because the establishment of high co-payments among people with chronic diseases could become a ‘tax on illness’ [6]. Accordingly, despite the fact that, on average, co-payments do not lead to deterioration in the health status of the population, there are two important exceptions: low-income people and chronic patients [7]. Therefore, the consequences of the introduction of drug co-payments could be worse among people with chronic or severe diseases than among other patients.

The 2012 Spanish reform, implemented by the Royal Decree Law 16/2012 (RDL 16/2012), increased the cost-sharing of both the working population and pensioners (with monthly contribution ceilings for pensioners). This reform established co-payments depending on income (i.e., according to three income intervals) but regardless of the nature of the disease to be treated (e.g., according therapeutic groups of drugs), with the exception of ATC drugs for the treatment of chronic diseases that had a reduced contribution, which had been introduced in 1978 [8]. However, this reform also increased the contribution ceiling per pack of ATC drugs with reduced contribution (cf. Table 1). For this reason, the main aim of this paper is to verify the heterogeneity of the effect of this cost-sharing change in pharmaceutical consumption by therapeutic groups to demonstrate the relevance and necessity of taking it into account. We evaluate the therapeutic groups that were most affected by the co-payment change. In this regard, we hypothesise, in accordance with existing evidence [9,10], that the pharmaceutical consumption will decrease as a result of the increase in the co-payment share. We also estimate the effect for those individuals who have been dispensed medicines from two or more therapeutic groups compared with the effect of those people who have been dispensed medicines from only one therapeutic group. This analysis could serve as a useful approach for the consumption of people with comorbidities. Thus, we hypothesise the decrease will be especially marked among people with several diseases because these patients are dependent on high levels of pharmaceutical private spending [11]. Moreover, we also estimate the monthly effect and hypothesise that the main effect of the reform on drug prescriptions will be mitigated during the following months as some studies have shown [12,13, 14].

**Table 1:** Coinsurance change.

		Before		After	
Groups and contribution codes created by SNHS	Income intervals (€)	Coinsurance (%)	Monthly ceiling (€)	Coinsurance (%)	Monthly ceiling (€)
Pensioners					
Non-contributory pensioners, toxic syndrome or disability (contribution code: TSI 001)	-	0	-	0	-
Low-income contributory pensioners (contribution code: TSI 002)	< 18,000	0	-	10	8
Middle-income contributory pensioners (contribution code: TSI 002)	18,000–99,999	0	-	10	18
High-income contributory pensioners (contribution code: TSI 005)	≥ 100,000	0	-	60	60
Working population					
Toxic syndrome; disability; recipients of income from social integration; unemployed who have used up their unemployment benefit as long as their situations persist; work accidents and occupational diseases (contribution code: TSI 001)	-	40		40/0	-
Low-income working population (contribution code: TSI 003)	< 18,000	40	-	40	-
Middle-income working population (contribution code: TSI 004)	18,000–99,999	40	-	50	-
High-income working population (contribution code: TSI 005)	≥ 100,000	40	-	60	-
ATC drugs with reduced contribution regardless of the contribution code of individuals	-	10	Ceiling per pack (€)	10	^NOTE^ Ceiling per pack (€)
			2.16		4.24
List of 426 medicines excluded from public provision	-	40, for working population and 0, for pensioners	-	100	-

NOTE: Despite the ceiling per pack in 2012 being € 4.24, it is updated automatically each January according to the changes in the consumer price index.

In order to avoid a biased interpretation of the reform’s impact on pharmaceutical consumption, it is quite important to take into account three key factors that may have impacted, together with the co-payment increase, on individuals’ consumption. First, the possibility of stockpiling after the reform’s announcement must be considered. To detect this, we investigate whether there was anticipated acquisition of drugs (i.e., stockpiling) for some therapeutic groups after the reform’s announcement. We hypothesise, according to the existing evidence, that the expected negative impact of the cost-sharing change on drug consumption was preceded by previous anticipated purchasing of drugs [15], especially, among those therapeutic groups related to chronic diseases [16]. Second, the RDL 16/2012 was a bundle policy that apart from the co-payment increase included other relevant measures among which we highlight the exclusion of 426 drugs from public provision on September 1^st^, 2012. In this regard, we hypothesise that this will have a sizable effect on the pharmaceutical consumption after the reform (as [13] suggested) and, consequently, the negative effect on consumption will be mitigated if we remove the list of medicines excluded from public provision. Third, there was some variation in the date for the implementation of monthly contribution ceilings. The effective implementation of monthly contribution ceilings was not done simultaneously in all the Autonomous Regions (e.g., on November 1^st^, 2012 in the Canary Islands). Thus, we hypothesise, without previous evidence of it, that in November 2012, a transitory recovery in pensioners' consumption took place as a consequence of the effective implementation of their contribution ceilings.

This study has been carried out in one of the seventeen Spanish Autonomous Regions, the Canary Islands, which are composed of seven islands (Tenerife, La Palma, La Gomera, El Hierro, Gran Canaria, Fuerteventura and Lanzarote). Each Autonomous Region has authority to organise and manage the provision and resources for health care services for its citizens. Nonetheless, the main policy on medical products, which includes the regulation of the co-payment system, is state-based rather than regional.

This research provides interesting contributions because we compare the reform’s impact among 13 therapeutic groups (with 13 price-elasticities of demand) composed of medicines used for the treatment of chronic (e.g., cardiovascular agents and anti-hyperlipidemics) and non-chronic (e.g., genitourinary and pulmonary drugs) illnesses, evidence of which is very limited in Spain. In this regard, we would like to place special emphasis on the relevance of our paper in terms of testing the heterogeneity of the effect of a change in pharmaceutical cost-sharing by therapeutic groups. Consequently, the existence of heterogeneity in the impact of medicines used for the treatment of chronic and non-chronic diseases would indicate that establishing co-payment according to therapeutic groups would be required. Additionally, we investigate the impact of the reform among patients with comorbidities, evidence about which is also quite limited. Finally, we provide a robust and innovative analysis, as we estimate the impact on pharmaceutical consumption controlling for three key factors (i.e., possibility of stockpiling, exclusion of 426 drugs from public provision and varied implementation dates of monthly contribution ceilings’).

### 1.1. International evidence of co-payment effects on consumption, utilisation or adherence by therapeutic groups

Two scientific publications [9,10] have collected some international evidence about the effect of drug co-payment and both found that pharmaceutical co-payments reduced the use of drugs. One study [17] with data from the Pharm-net database (between 1999 and 2005) analysed the influence of co-payment on two commonly used medicine groups (cholesterol-lowering medication and acid-blocking agents) in Belgium. The study (using change-point linear mixed models) revealed that an increase in cost sharing reduced the use of medications in the short term.

#### 1.1.1. Stockpiling evidence

Regarding empirical evidence of stockpiling, there was a study [16] for Spain in which the authors estimated the association between the implementation of €1 co-payment per prescription and drug consumption of the publicly insured population of Catalonia. Using administrative data (between 2012 and 2014) on monthly pharmaceutical consumption (measured in Defined Daily Dose (DDDs)), they observed that patients anticipated drug purchasing during the two months prior to the reform. They also found that pensioners stockpiled more than the working population and by therapeutic groups, they uncovered that this anticipation effect is more common among those therapeutic groups related to chronic diseases.

Another study [18], using data from the entire Danish population in 2000 (with data from the Danish Medicines Agency), focused on two specific drugs: insulin, with foreseeable future need, and penicillin for acute infections, with unforeseeable future need. The authors demonstrated (through discontinuous regression models) that the amount of insulin increased at the moment of the cost-sharing’s announcement, giving rise to a stockpiling phenomenon. There is an update on this previous study [19], which centred on two different antibiotics: penicillin and dicloxacillin, whose future need is impossible to predict and, therefore, it is not possible to stockpile before a cost-sharing increase. The authors concluded that the increase in prescription co-payment was quite price inelastic.

#### 1.1.2. Effect of co-payments in essential (related to serious illnesses) and less essential medicines

Another overall conclusion of the literature on co-payment is that the demand for essential medicines, which are related to serious illnesses, is more inelastic than the demand for less essential medicines, which are related to less serious illnesses. In this regard, we highlight the following four studies.

The first study [20] applied data from both 1994–95 and 1996–97 National Population Health Surveys (NPHS) in Canada for seniors (65–84 years old) and people that required social assistance. The authors analysed the effect of prescription drug insurance coverage status on the use of specific medications (grouped by chronic and acute diseases; and by essential and less essential medications). The study found (by the use of instrumental variables method) that drug charges did not affect the use of drugs for either chronic medications or essential drugs but had some impact on the use of drugs for acute conditions and less essential medications.

The second study [21] used four provincial health databases in Canada (1996) to estimate the impact of introducing drug co-payment for the elderly and welfare recipients on essential and less essential medications. The authors concluded (through random-effects, pooled-time series regression) that the co-payment change reduced the use of less essential drugs more than the use of essential ones.

Regarding the third study [22], the authors applied a register-based data set from a 20% random sample of the population (between 2000 and 2003) in Denmark. They investigated the price sensitivity of demand for specific prescription drugs comparing the use of essential medicine with other less essential drugs. The study reflected (by the use of a regression kink design) that essential drugs had much lower associated average price sensitivity than other less essential medicines.

Finally, the fourth analysis [23] used interrupted time-series data collected by the Drug Utilization Sub-committee (DUSC) of the Pharmaceutical Benefits Advisory Committee (PBAC) in Australia (1990). The authors detected (through segmented linear regression models) that the cost-sharing change reduced the use of essential and non-essential medicines. Nevertheless, there also was a post-intervention trend for increased prescriptions of essential drugs after the initial decline, which was not detected for non-essential medicines.

Overall, the literature tends to conclude that people are more sensitive to cost-sharing changes with less essential than with essential medicines, and also there is evidence of higher sensitivity among acute patients to cost-sharing changes than chronic patients (with demonstrated needs). The following studies are examples of this.

#### 1.1.3. Effect of co-payments in patients with acute and chronic conditions

The authors of the following study [24] applied data from claims and eligibility records of Medicare Part D members that did not receive the low-income subsidy in the United States (between 2009 and 2011). They analysed the effect of a cost-sharing reduction (from $35 to $0-$4 per month of treatment) on brand-name statins (as generic statins maintained cost-sharing, their consumers were used as control group) and concluded (by the use of a logistic regression model) that the reduction in co-payment statins improved the adherence to brand name statins.

Another paper [25] used data from the Federfarma to analyse the effect of co-payments on the use of statins in Italy (between 2001 and 2007). The control group were consumers in regions without co-payments for statins, while the intervention group were consumers in regions with co-payment for statins. The paper inferred (through segmented regression analysis) that co-payment slightly reduced the consumption trend but not the level of consumption.

The following study [26] applied longitudinal administrative micro-data to analyse the effect of a change in cost-sharing in Canada (between 1995 and 1997) for senior chronic patients. The results showed (using an instrumental variable method) low expenditure elasticity (between -0.12 and -0.16). Also, we found two studies [27,28] using administrative databases of the Canadian Ministry of Health to assess the impact of a drug cost-sharing increase on the use of drugs among seniors with rheumatoid arthritis in Canada (between 1996 and 2002). The authors of both papers concluded (through instrumental variables and difference-in-differences design, respectively) that despite the demand for prescription drugs decreasing, the demand for physician visits increased.

One final paper [29] used the database from the Swedish Prescribed Drug Register to study how refill adherence varies according to patients’ co-payment levels for antiepileptic drugs in Sweden (2007). The study reflected (by the use of multilevel mixed-effects linear regression models) that the adherence rate was greater for antiepileptic drugs among those patients with free full-coverage than for those patients that had to pay 100% of the drug price.

### 1.2. The Royal Decree Law 16/2012 in Spain

The Spanish Government implemented the RDL 16/2012, which is the most important Health System reform in the last forty years, in order to control public health spending [30,31] and with the aim of achieving a long-term sustainable Spanish National Health System (SNHS) [30]. This reform, in the form of bundle policy, included some measures among which we highlight:

First, the working population went from 40% cost-sharing regardless of income to a co-payment percentage depending on three different income intervals. Similarly, the majority of pensioners went from free full-coverage to 10% co-payment with monthly ceilings (cf. Table 1). It is of interest that not all the Autonomous Regions applied the monthly contribution ceilings simultaneously (e.g., on November 1^st^, 2012 with the introduction of a computer system in the Canary Islands). To recover the money paid between July 1^st^, 2012 and November 1^st^, 2012, pensioners had three alternatives to calculate the extra money that they had paid over their monthly contribution ceilings: convert it into an unused balance; to receive payments into their current account every six months until the extra money paid was compensated; or to make an application at the request of stakeholders [32].

Second, the proposed implementation of an electronic prescription system that would help reduce stockpiling and more easily control all the relevant information (e.g., medical history, treatments, medicines prescribed, adherence to treatments and prices) [33]. Despite this reform (RDL 16/2012) further regulating the establishment of an electronic prescription system, there had previously been other laws that had the same purpose: Law 16/2003; Law 29/2006; and RDL 1718/2010, which detailed general aspects for the development of electronic prescriptions and the right way to successfully implement it. The implementation of the electronic prescription system in all Canary Island municipal districts was a progressive process that was fully implemented in Spain in about 2015 [33]. In fact, in those areas where electronic prescriptions were fully implemented, it was still possible for the doctor to choose between the traditional and the electronic prescription system [34]. This absence of prescription control facilitated prescription duplication between 2011 and 2013 (the period of our study) and made it possible to stockpile drugs. For this reason, it makes sense to analyse the existence of the stockpiling in this study.

Third, there was the exclusion from public provision of 426 drugs on September 1^st^, 2012. The reasons for the exclusions were: the establishment of selected prices; the coexistence with an over-the-counter medication with which it shared the active ingredient and dosage; the consideration of the medication as advertising in the European environment; the favourable safety and efficacy profile of the active ingredient; the excluded drug was indicated in the treatment of minor symptoms; or the drug fulfilled any of the criteria of non-inclusion in public financing specified in section 2, Article 89, RDL 16/2012 [30].

### 1.3. Evidence of RDL 16/2012 effects on consumption, utilization or adherence by therapeutic groups

Several studies that analysed the impact of RDL on pharmaceutical dispensed prescriptions agree that the reform generated an immediate reduction in pharmaceutical prescriptions [12,35,36,37,38,39]. Nevertheless, some studies found this reduction temporary [12,13,14]. Despite the majority of these contributions only showing a general effect, we have found four papers that analysed the impact of the reform on specific drugs.

The first study [12] used the IMS Health database (between 2012 and 2015). The analysis revealed (through segmented linear regression models) that the reform generated an immediate non-permanent reduction in prescription consumption. After the first 12–18 months, consumption levels recovered. By therapeutic groups (anti-diabetics; antithrombotic; and asthma and COPD drugs), the authors detected that those medications prescribed for chronic diseases (e.g., diabetes) were less sensitive to changes in prices than those prescribed for less serious diseases. The estimated variation rate of the DDDs was negative but decreased for three therapeutic groups in the following 6, 12, 24 and 38 months following the reform.

The evidence of stockpiling is quite limited in Spain. In fact, [12,13] have been the only studies found that analysed stockpiling effects caused by the RDL 16/2012. The authors showed that a stockpiling effect took place between the reform’s announcement (April 20^th^, 2012) and its implementation (July 1^st^, 2012). In particular, the elderly group is more likely to employ this strategic behaviour because the elderly (and the rest of pensioners) benefited from free full coverage until the arrival of RDL 16/2012, which generated a problem related to the ‘Moral Hazard’ [40–42]. Moreover, the elderly have a natural tendency to suffer more illnesses and, consequently, consume more drugs than younger people [12]. Nevertheless, there has been no evidence reported as to which therapeutic groups practise stockpiling the most.

Regarding the second research study [13], the authors used aggregate monthly data from the Official Association of Pharmacists of the region of Murcia (between 2008 and 2013). The analysis showed (through segmented regression analysis by therapeutic groups) that co-payment changes affected the consumption of drugs in the short term. However, this negative effect, slowed down, and was even compensated for gradually. Some groups of medicines showed reduced consumption more than others (e.g., the use of benzodiazepines even increased).

With respect to the third study [14], the authors applied data from several electronic information systems of the Valencia Health System to analyse the impact of pharmaceutical co-payment on adherence to essential medication in patients with acute coronary syndrome in the region of Valencia (between 2009 and 2013). This study showed (through the Difference-in-Differences method) that the cost-sharing change generated a temporary reduction in adherence among the middle-income working population and low-income pensioners for essential medications with high prices.

We also found a fourth paper [31] demonstrating that the implementation of new co-payments led to a 10% reduction in the volume of units sold in the following three months after the implementation of the reform. This paper made reference to a report from the pharmaceutical industry [43], which reflected that the decrease experienced in pharmaceutical prescriptions was not homogeneous across drugs. Those medications indicated for the treatment of chronic illnesses fell more than those for acute diseases.

Finally, despite the following study [16] not exactly analysing the reform of July 2012, we consider it worth including in this section because the reform analysed in this paper was implemented in a Spanish region (i.e., publicly insured population of Catalonia) in June 2012. Specifically, the authors tested the association between the implementation of €1 co-payment per prescription and drug consumption, and they observed that a uniform capped low co-payment may considerably reduce drug consumption (measure in DDDs) among individuals who previously benefited from free prescriptions.

## 2. Methodology

### 2.1. Ethics statements

The Canary Islands Health Service provided us with the data. In this regard, our study complied with the terms of service for the Canary Islands Health Service database and we used these data with appropriate permissions from this third party. In addition, we also clarify that all data were anonymized before we accessed them for this study.

### 2.2. Data

We used a representative random sample (provided by the Canary Islands Health Service) of 40,471 people covered by the SNHS in the Canary Islands, stratified into seven health areas and labour status (working population or pensioners). For each individual, there is information about dispensed medications prescribed by the SNHS from one year before (2011) to one year after (2013) the implementation of the RDL 16/2012 on July 1^st^, 2012. The resulting dataset included information on individuals (i.e., age, sex, island of residence, health centre, simulated income, working population or pensioner status, contribution code and contribution ceiling); information about any change in individual circumstances during the period under study (e.g., moving from working population to pensioner or changes in income); and prescription information (i.e., prescription date, daily number of prescriptions per individual, daily pharmaceutical expenditure per individual, National Code (NC), anatomical, therapeutic and chemical code (ATC) and Number of DDDs). From these disaggregated data, we computed, monthly number of dispensed prescriptions and monthly pharmaceutical expenditure for each patient. In total, there are 24 observations per individual, one for each month of the studied period. For those months without consumption by individuals we recorded a zero value.

The original database did not include information about the specific income of each individual of the sample but just which of three income intervals established by the SNHS the individual belongs to (cf. Table 1). For this reason, we built a simulated income variable. To create it, we ran 10,000 Monte Carlo simulations for the specific income of each individual as follows. First, we specified set seed 12345 to set the seed of the random-number generator so that the results would be reproducible. Second, to obtain 10,000 estimates, we created a place in memory (by the use of *postfile buffer* command) where we stored the results. Third, we ordered that the process should be repeated 10,000 times (by the use of *forvalues i = 1/10*,*000* command). Fourth, we dropped the previous data (by the use of *quietly drop all* command) and generated the five simulated income intervals (I<€6010; 6,010 ≤ I ≤ €12,020; 12, 020 ≤ I ≤ €18, 030; 18, 030 ≤ I ≤ €21,035; I > €21,035) from a random uniform distribution. These intervals are the same as those used by the Spanish Tax Agency to apply Personal Income Tax. We ordered here that in the case of those individuals with income below €18,000 (as in the original SNHS interval), the income must fall within one of the first three new intervals. Likewise, in the case of those individuals with income between 18,000 and €100,000, the income must fall within the two last new intervals. Also, for generating this simulated variable, we controlled the percentage of people that belonged to each one of the above five income intervals. We obtained this information from a Spanish Tax Agency database for 2013. This database disaggregated this information by municipal district. As we had the municipal district variable in our database, we used it as a way of merging between each database. After generating the simulated income variable, we estimated the mean and stored the estimations (by the use of *post buffer* command). Finally, we wrote the results (by the use of *postclose buffer* command) in our database.

As our database did not contain the number of DDDs variable, we built it for each one of the 8,121 drugs that compose the database by creating a complementary one, using ‘National’ and ‘ATC’ codes. To obtain this variable, we used the DDDs established by the World Health Organization (WHO) according to the active ingredient of each drug. This information is available on the WHO Collaborating Centre for Drug Statistics Methodology website [44]. In the cases that this website did not provide the necessary information, we used a report from the Spanish Ministry of Health, Equality and Social Policy [45]. Then, we obtained both, the number of doses contained within each drug and the quantity of active ingredient that each dose contains through the Spanish Agency of Medicines and Medical Devices website [46]. Finally, we calculated the number of DDDs:

$$No.DDDs=\frac{No.doses\;contained\;in\;each\;drug\;pack\times qty.active\;ingredient\;contained\;in\;each\;dose}{DDDs\;established\;by\;the\;WHO\;according\;to\;the\;active\;ingredient\;of\;each\;drug}$$

In this regard, as WHO Collaborating Centre for Drug Statistics Methodology website specifies, most products that belong to the dermatological therapeutic group are for topical use. Accordingly, no DDDs are assigned because the amount given per day can vary greatly according to the intensity and distribution of the disease. For this reason, for topical dermatological preparations, instead of using the DDDs, it was expressed in grams of preparation regardless of strength. Then, with this information (grams of preparation), the procedure to calculate the number of DDDs for each topical dermatological drug was the same as for the rest of the drugs.

$$No.derma.DDDs=\frac{No.doses\;contained\;in\;each\;drug\;pack\times qty.active\;ingredient\;contained\;in\;each\;dose}{grams\;of\;preparations\;regardless\;of\;strength}$$

We used quasi-experimental difference-in-differences design to analyse the change in drug consumption (measured in monthly number of DDDs per individual) caused by the reform implemented on July 1^st^, 2012. The database included two variables that code different medicines: the National Code of medicine (NC, with 7,799 different codes included in the database), which identifies drugs with the highest disaggregated level; and the Anatomical, Therapeutic, Chemical classification system (ATC, with 357 different codes included in the database), which aggregates drugs in families of medicines with similar anatomical, therapeutic and chemical characteristics. With the aim of having a more aggregated level of therapeutic groups for the analysis, we used the ATC variable to create 15 different therapeutic groups. Each one of these groups has an associated price-elasticity according to the study by Puig Junoy J. et al. [5], in which the authors calculated different price-elasticity through a difference-in-differences approach. Price-elasticity varied from -0.26 to -0.03 depending on therapeutic group. We could not calculate our own price-elasticity because of limitations in the database (i.e., the variability of co-payments was quite low (from 0 to 10% or from 40% to 50%) and the contributions of the users in the database were very small). There is a study [4] that estimated a price-elasticity (for the elderly population) around -0.20 for non-chronic and around -0.08 for chronic diseases. These values were very useful, as a guide, to interpret our results. We had to remove from the sample Biological and immunological agents because the number of observations that belonged to these therapeutic groups was too small. Therefore, the analysis only focused on the 13 remaining therapeutic groups.

### 2.3. Sample

We defined one control and two intervention groups. Our control group was low-income working population (N_C_ = 25,588), subject to 40% co-payment throughout the period and, therefore, not affected by the reform (except for some ATC levels: ATCs with reduced contribution for some chronic diseases, which increased their contribution ceiling and medicines withdrawn from public provision in September 2012 (cf. Table 1)). This group, whose contribution code is TSI 003 (cf. Table 1), is composed of people with annual incomes lower than €18,000 and aged between 16 and 65 and their dependents (mainly minor children or minors in foster care). In addition, this group includes children and pregnant women (during the pregnancy, birth and postpartum periods) from countries without collaboration agreements with Spain or immigrants. Finally, working population from a different Autonomous Region in which the prescription does not indicate the contribution code assigned, are also included in this group (only while the electronic prescription system was not fully implemented in all the Autonomous Regions). It should be clarified that, people with toxic syndrome, the disabled working population, recipients of income from social integration, unemployed who had used up their unemployment benefit, and those off work because of work accidents or occupational diseases are not included in the working population group (TSI 003) because they have a specific contribution code (TSI 001) assigned (cf. Table 1).

Our first intervention group is low-income pensioners (N_I1_ = 7,898), who moved from free full coverage to 10% co-payment with a monthly ceiling for all medications of €8. This group, whose contribution code is TSI 002 (cf. Table 1), refers to people with annual incomes lower than €18,000 over the age of 65 (who have reached retirement age) and those under the age of 65 (who have taken early retirement). Dependents of low-income pensioners (mainly children or minors in foster care, e.g., grandchildren) also belong to this group. It is of interest that, non-contributory people with toxic syndrome and disabled pensioners are not included in the pensioner group (TSI 002) because they have a specific contribution code (TSI 001) assigned (cf. Table 1).

Our second intervention group is middle-income working population (N_I2_ = 6,985), who moved from 40% to 50% co-payment. This group, whose contribution code is TSI 004 (cf. Table 1), includes people with annual incomes between €18,000 and € 99,999 and aged between 16 and 65 and their dependents (mainly minor children or minors in foster care). This group also includes foreigners with European Health Insurance Cards and people from countries with collaboration agreements with Spain that does not prove their status as pensioners.

### 2.4. Empirical model

We defined the linear regression model:

$$Y_{igt}=\beta_{0}+\sum_{t^{\prime }\neq 0}\beta_{t^{\prime }}D_{t^{\prime }}+\delta I_{gt}+\gamma I_{gt}D_{t\geq 1}+\mu C_{igt}+\varepsilon_{igt}$$

where i indexes individual, g indexes group, and t indexes time. *Y*_*igt*_ is monthly pharmaceutical consumption, measured as number of monthly DDDs per individual of the sample. *D*_*t*_ is a monthly dummy variable used to control for time trends affecting all groups (1, for July, 2011; 2, for August, 2011,…, and 24, for June, 2013). *I*_*gt*_ is a measure of the intervention, it is a three-level categorical variable for one control and two intervention groups: control group is low-income working population (0) and intervention groups are low-income pensioners (1) and middle-income working population (2). *D*_*t*≥1_ is the policy break indicator, three-level categorical variable: before reform’s implementation and before its announcement (0), before the reform’s implementation but after its announcement (1), after the reform’s implementation (2). *C*_*igt*_ is a vector of individual-specific covariates, including age, sex, municipal district and simulated incomes. *ε*_*igt*_ is the random error. We computed the analysis by the use of Stata-15.

We estimated a total of 16 regression models adjusted for clustering by individual. We first considered all the therapeutic groups (general analysis: $Y_{igt}^{1}$ = number of monthly DDDs per individual for model 1). We then estimated thirteen linear regression models, one for each therapeutic group ($Y_{igt}^{2}$ = number of monthly cardiovascular DDDs per individual for model 2; $Y_{igt}^{3}$ = number of monthly anti-hyperlipidemics DDDs per individual, for model 3,…, $Y_{igt}^{14}$ = number of monthly pulmonary drugs DDDs per individual, for model 14). We finally estimated two linear regression models: the fifteen, for estimating the behaviour of individuals with comorbidities ($Y_{igt}^{15}$ = number of monthly DDDs per individual who each month was dispensed medicines from two or more therapeutic groups, for model 15) and the sixteen for estimating the behaviour of individuals with one disease ($Y_{igt}^{16}$ = number of monthly DDDs per individual who each month was dispensed medicines from one therapeutic group, for model 16). We should highlight that we actually do not have information on patients’ comorbidities (as our database does not provide information about the specific diseases of individuals). We have also assumed that the approach of comorbidities among individuals with different diseases from the same therapeutic group (e.g., high blood pressure and bad cholesterol) cannot be considered in the analysis. Therefore, we have approached patients having comorbidities as those who have been dispensed medicines from two or more therapeutic groups.

For each regression model, we estimated both, the period between the RDL’s announcement and implementation–from May 1^st^, 2012 to July 1^st^, 2012 –and the period after the RDL’s entry into force–from July 1^st,^ 2012 to June 1^st^, 2013–. Despite the reform’s announcement taking place on April 20^th^, 2012, we decided to analyse the period between the RDL’s announcement and implementation as of May 1^st^, 2012 in order to start the analysis with a whole month. As the regression models were composed of one control and two intervention groups, we obtained two different DID coefficients for each model: one for the difference between the control and the first intervention group, and another for the difference between the control and the second intervention group. Apart from this, there was a monthly analysis that included the estimates for each month of the studied period. Each coefficient showed the change in the number of monthly DDDs per individual.

## 3. Results

### 3.1. Descriptive statistics

Low-income working population is the most numerous group in the sample followed by middle-income working population and low-income pensioners. The percentage of women is greater among low-income pensioners (58.4%) and low-income working population (52.6%). As for demographic information, there are fewer pensioners in Fuerteventura, Lanzarote and Tenerife than in the rest of the islands where the percentage remains at around 20%. The majority of low-income pensioners were dispensed medicines for the treatment of cardiovascular drugs (49.9%). Regarding low-income working population, the most common drugs dispensed to this group were those indicated for the treatment of analgesic diseases (8.4%). With respect to middle-income working population, the majority of them were dispensed medicines for the treatment of cardiovascular diseases (9.8%). Also, the individuals who belonged to this group were those who recorded more changes in their contribution code after the reform, moving to low-income working population. It is of interest to highlight that the group that seemed to include more patients with comorbidities was the low-income pensioners (cf. Table 2).

**Table 2:** Demographic key outcomes variables.

Low-income Pensioners (Intervention 1)			Low-income working population (Control)		Middle-income working population (Intervention 2)	
*% of individuals*						
19.5 (7,898)			63.2 (25,588)		17.3 (6,985)	
*Age average of individuals within each group*						
64			32		34	
*% of individuals within each group by sex*						
Women		Men	Women	Men	Women	Men
58.4		41.5	52.6	47.4	49.0	51.0
*Income average of individuals within each group*						
8889			8880		52094	
*% of individuals per island*						
Fuerteventura		10.8	71.9		17.3	
Gran Canaria		22.9	59.6		17.49	
La Gomera		21.9	61.3		16.8	
El Hierro		23.0	60.0		17.1	
Lanzarote		12.9	69.9		17.2	
La Palma		23.2	60.3		16.5	
Tenerife		18.4	64.4		17.2	
*% of individuals within each group that have obtained medicines at any time during the studied period*						
Cardiovascular agents		49.9	7.3		9.8	
Antihyperlipidemics		31.0	4.1		5.5	
Endocrine/metabolic agents		44.2	6.2		7.0	
Central nervous system agents		39.0	7.7		7.8	
Biological*		0.4	0.2		0.3	
Diabetes drugs		17.7	2.3		3.0	
Dermatological		6.7	2.0		1.84	
Gastrointestinal drugs		4.57	1.0		0.9	
Analgesics/anti-inflammatories		33.6	8.4		7.4	
Eye, ear, nose and throat preparations		9.0	0.7		1.0	
Anti-infective		7.3	5.6		5.2	
Immunological agents*		0.0	0.0		0.1	
Upper respiratory agents		1.7	1.0		1.2	
Genitourinary agents		9.3	1.8		1.9	
Pulmonary drugs		11.8	5.2		5.1	
*% of individuals who have been dispensed medicines from two or more therapeutic groups*						
54.05 (4,562)			35.61 (3,006)		10.34 (873)	
*% of individuals who have been dispensed medicines from one therapeutic group*						
10.42 (3,336)			70.50 (22,582)		19.08 (6,112)	
*Number of individuals that changed their contribution code after the reform*						
	To:	Low-income pensioners (TSI 002)	Low-income working population (TSI 003)		Middle-income working population (TSI 004)	
From:	Low-income pensioners (TSI 002)	-	(350)		(9)	
	Low-income working population (TSI 003)	(383)	-		(938)	
	Middle-income working population (TSI 004)	(136)	(1,070)		-	

The number of individuals is shown in parentheses.

*Biological and immunological agents have been removed from the sample because the number of individuals in each of these groups is not enough.

The therapeutic groups are sorted by price-elasticity (at the top the most inelastic, while at the bottom the most elastic).

The average monthly pharmaceutical prescriptions per individual was much higher in all the therapeutic groups for pensioners than for the working population during the three different analysed periods (9 months before the announcement of the reform; 2 months during the reform’s announcement and its implementation; and 11 months after the reform’s implementation) (cf. Table 3).

**Table 3:** Average monthly pharmaceutical prescriptions per patient before, during and after the reform announcement and variation rates.

	9 months before (August 1^st^, 2011-May 1^st^, 2012)	2 months during (May 1^st^, 2012-July 1^st^, 2012) ^NOTE 1^	11 months after (July 1^st^, 2012-June 1^st^, 2013)	V. rate: consumption ^NOTE 2^ (%)	V. rate: price ^NOTE 2^ (%)	V. rate consumption (%)/V. rate price (%)
**All groups**					** **	** **
Low-income pensioners	5.51 (435,789)	6.25 (98,675)	5.34 (506,539)	-9.18	9900	-0,000927273
Low-income working population	0.77 (196,868)	0.84 (42,998)	0.94 (287,904)	16.77	0	-
Middle-income working population	0.83 (57,637)	0.91 (12,711)	1.04 (86,903)	19.54	25	0.78
**Cardiovascular agents**					** **	** **
Low-income Pensioners	1.11 (88,054)	1.23 (19,513)	1.07 (101,751)	-8.55	9900	-0,000863636
Low-income working population	0.11 (26,881)	0.12 (6.193)	0.12 (37.645)	4.35	0	-
Middle-income working population	0.14 (9,741)	0.16 (2,243)	0.17 (14,083)	13.33	25	0.53
**Anti-hyperlipidemics**					** **	** **
Low-income Pensioners	0.37 (29,293)	0.42 (6,666)	0.38 (36,030)	-3.80	9900	-0,000383838
Low-income working population	0.04 (10,825)	0.05 (2,550)	0.05 (15,946)	11.11	0	-
Middle-income working population	0.06 (4,124)	0.07 (988)	0.07 (5,915)	7.69	25	0.31
**Endocrine/metabolic agents**					** **	** **
Low-income Pensioners	0.65 (51,483)	0.74 (11,752)	0.61 (58,083)	-12.23	9900	-0,001235354
Low-income working population	0.07 (17,369)	0.08 (4,013)	0.08 (24,668)	6.67	0	-
Middle-income working population	0.08 (5,495)	0.09 (1,250)	0.09 (7,790)	5.88	25	0.24
**Central nervous system agents**					** **	** **
Low-income Pensioners	0.91 (71,779)	1.03 (16,324)	0.97 (92,312)	0.00	9900	0
Low-income working population	0.12 (32,679)	0.14 (7,361)	0.16 (50,320)	23.08	0	-
Middle-income working population	0.12 (8,563)	0.14 (1,965)	0.17 (13,994)	30.77	25	1.23
**Diabetes drugs**					** **	** **
Low-income Pensioners	0.29 (22,646)	0.33 (5,219)	0.30 (28,751)	-3.23	9900	-0,000326263
Low-income working population	0.03 (8,441)	0.04 (1,991)	0.04 (12,771)	14.29	0	-
Middle-income working population	0.04 (2,927)	0.05 (684)	0.05 (4,488)	11.11	25	0.44
**Dermatological**					** **	** **
Low-income Pensioners	0.10 (8,089)	0.12 (1,908)	0.09 (8,453)	-18.18	9900	-0,001836364
Low-income working population	0.02 (5,565)	0.02 (1,222)	0.03 (8,286)	50.00	0	-
Middle-income working population	0.02 (1,337)	0.02 (314)	0.03 (2,204)	50.00	25	2
**Gastrointestinal drugs**					** **	** **
Low-income Pensioners	0.06 (4,967)	0.07 (1,145)	0.07 (6,211)	7.69	9900	0,000776768
Low-income working population	0.01 (2,686)	0.01 (594)	0.01 (3,890)	0.00	0	-
Middle-income working population	0.01 (656)	0.01 (140)	0.01 (1,062)	0.00	25	0
**Analgesics/anti-inflammatories**					** **	** **
Low-income Pensioners	0.70 (55,277)	0.80 (12,724)	0.67 (63,202)	-10.67	9900	-0,001077778
Low-income working population	0.11 (27,320)	0.11 (5,665)	0.13 (41,431)	18.18	0	-
Middle-income working population	0.09 (6,373)	0.10 (1,331)	0.12 (9,893)	26.32	25	1.05
**Eye, ear, nose and throat preparations**					** **	** **
Low-income Pensioners	0.18 (14,545)	0.22 (3,474)	0.11 (9,995)	-45.00	9900	-0,004545455
Low-income working population	0.01 (2,300)	0.01 (583)	0.01(2,697)	0.00	0	-
Middle-income working population	0.01 (977)	0.02 (227)	0.01 (1,028)	-33.33	25	1.33
**Anti-infective**					** **	** **
Low-income Pensioners	0.08 (6,681)	0.09 (1,360)	0.14(12,946)	64.71	9900	0,006536364
Low-income working population	0.07 (16,693)	0.06 (3,226)	0.09(27,703)	38.46	0	-
Middle-income working population	0.06 (4,041)	0.06 (796)	0.09 (7,459)	50.00	25	2
**Upper respiratory agents**					** **	** **
Low-income Pensioners	0.02 (1,510)	0.02 (320)	0.02 (1,741)	0.00	9900	0
Low-income working population	0.01 (2,531)	0.01 (448)	0.01 (3,347)	0.00	0	-
Middle-income working population	0.01 (823)	0.01 (176)	0.01 (1,224)	0.00	25	0
**Genitourinary agents**					** **	** **
Low-income Pensioners	0.11 (9,002)	0.13 (2,005)	0.12 (11,621)	0.00	9900	0
Low-income working population	0.02 (3,996)	0.02 (1,090)	0.03 (8,361)	50.00	0	-
Middle-income working population	0.02 (1,270)	0.02 (293)	0.03 (2,434)	50.00	25	2
**Pulmonary drugs**					** **	** **
Low-income Pensioners	0.23 (18,111)	0.25 (4,002)	0.20 (18,788)	-16.67	9900	-0,001683838
Low-income working population	0.08 (18,788)	0.07 (3,662)	0.08 (23,260)	6.67	0	-
Middle-income working population	0.08 (5,335)	0.07 (994)	0.08 (6,421)	6.67	25	0.27

NOTE 1: It should be remembered that the reform’s announcement took place on April. 20^th^ 2012, but in order to consider a whole month, the analysis was started from May 1^st^, 2012. The therapeutic groups are sorted by price-elasticity (at the top the most inelastic, while at the bottom the most elastic).

NOTE 2: The variation rate was calculated comparing the average monthly pharmaceutical prescription per individual 11 months before the implementation of the reform with the average monthly pharmaceutical prescription per individual 11 months after the implementation of the reform. As low-income pensioners moved from 0% to 10% of co-payment it was not possible to obtain its exact price variation rate. For that, we have made an approach assuming that low-income pensioners moved from 0.1% to 10% (instead of from 0% to 10%).

(number in brackets)

**Fig 1:**
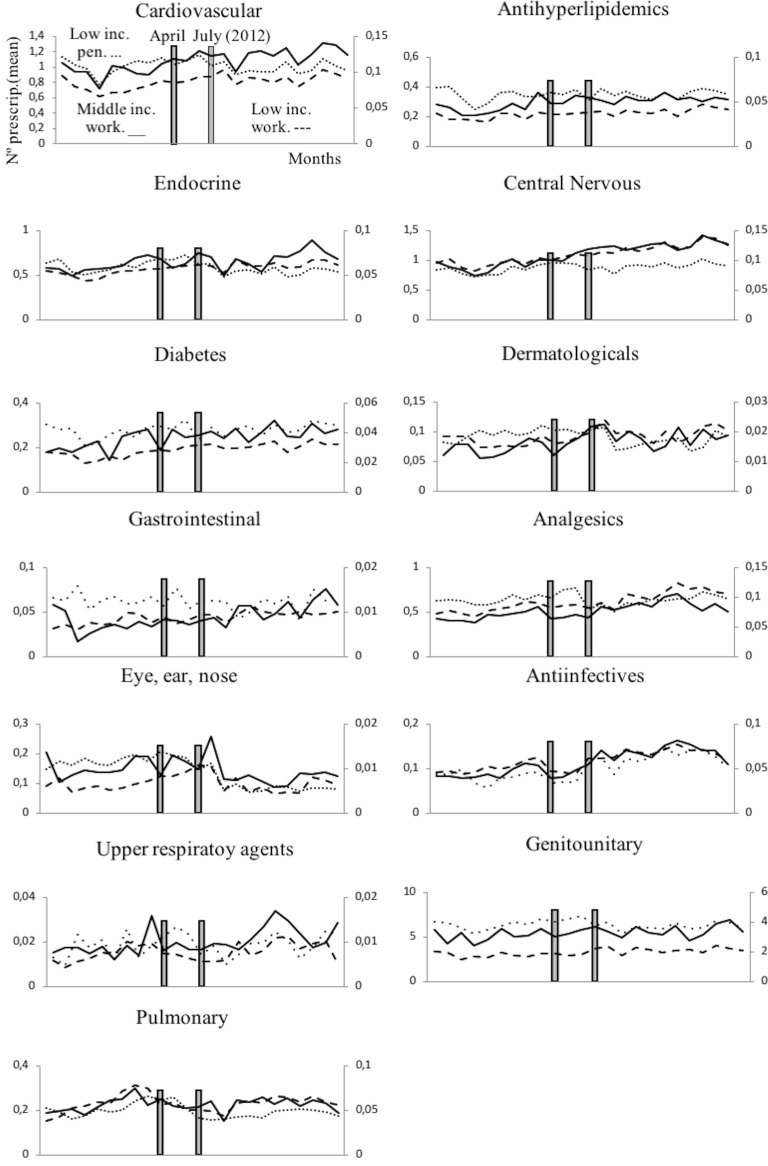
Monthly pharmaceutical consumption by therapeutic groups. The monthly period covers from July 2011 to June 2013; April 2012: April 20th, 2012 was the date on which RDL 16/2012 was announced; July 2012: July 1st, 2012 was the date on which RDL 16/2012 entered into force (date of cost sharing change). The therapeutic groups are sorted by price-elasticity (on the left the most inelastic, while on the right the most elastic). . . . . . . line corresponds to low-income pensioner population (first intervention group); ___ corresponds line to middle-income working population (second intervention group); -—line corresponds to low-income working population (control group).

The consumption variation rates showed that the only group that reduced its pharmaceutical consumption after the reform’s implementation was low-income pensioners (with a reduction of between 3.23% and 18.18%, except for eye, ear, nose and throat medication which showed a 45% decrease). The other individuals of the sample (low-income working population and middle-income working population) maintained their increasing consumption trend that existed before the reform (cf. Table 3 and Fig 1). We compared the consumption variation rate and price variation rate and we obtained that, on the whole, the effect of the reform on consumption was small (consumption variation rate/price variation rate was <1), except in the cases of central nervous system (1.23), dermatological (2), analgesics/anti-inflammatories (1.05), eye, ear, nose and throat preparations (1.33), anti-infective (2) and genitourinary agents (2), where there was a positive impact on pharmaceutical consumption among middle-income working population.

In the case of low-income pensioners, average monthly pharmaceutical prescriptions were higher in the period prior to the reform than after it for Cardiovascular agents, Endocrine/metabolic agents, Dermatological, Analgesics/anti-inflammatories, Eye, ear, nose and throat preparations, Upper respiratory agents and Pulmonary drugs. By contrast, there were more prescriptions after the reform for Anti-hyperlipidemics, Central nervous system agents, Diabetes drugs, Gastrointestinal drugs, Anti-infective and Genitourinary agents (cf. Fig 1).

The results also suggest the existence of stockpiling by pensioners in all therapeutic groups (except gastrointestinal drugs, anti-infective and upper respiratory agents), because they increased their average monthly pharmaceutical prescriptions after the announcement and reduced it after the reform. Nevertheless, this anticipation effect was not clearly detected among working population (cf. Table 3 and Fig 1).

### 3.2. Model estimates

The general analysis after the reform’s implementation reflected different results in consumption (measured as number of monthly a DDDs per individual) for each intervention group. While, low-income pensioners showed a decrease (-13.94), middle-income working population showed an increase (1.22) in the number of monthly DDDs per individual (cf. Table 4).

**Table 4:** Overall effect of the cost-sharing change on the pharmaceutical consumption.

	Low-income pensioners and low-income working population analysis	Middle-income working population and low-income working population analysis
**All groups**		
During-before the announcement	16.60*** (0.86)	0.58 (0.36)
After-during the announcement	-13.04*** (0.79)	1.22*** (0.33)

The table contains Difference-in-Differences estimates from linear regression models with robust standard errors. Each cell contains results of the model from different therapeutic groups. All regressions include age and age2, and time dummies. Within each cell, we first report the estimated coefficients; we then report in parentheses robust standard errors. The therapeutic groups are sorted by price-elasticity (at the top the most inelastic, while at the bottom the most elastic).

Significance levels

***p < 0.01

**p < 0.05.

By therapeutic groups, we also detected mixed results after the reform’s implementation. For low-income pensioners, we found statistically significant decreases in consumption for cardiovascular agents (-2.27), eye, ear, nose and throat preparations (-1,88), endocrine/metabolic agents (-1.31), analgesics/anti-inflammatories (-0,86), dermatological (-0,69), pulmonary drugs (-0.53) and genitourinary agents (-0,20). The results for central nervous system agents (1.01) and anti-infective drugs (0.24) reflected an increase in the number of monthly DDDs per individual. For middle-income working population, the majority of effects were not statistically significant and those statistically significant presented an increase in the number of monthly DDDs per individual after the reform’s implementation for cardiovascular agents (0.33), dermatological (0.12), anti-infective (0.06) (cf. Table 5).

**Table 5:** Overall effect of the cost-sharing change on the pharmaceutical consumption by therapeutic groups.

	Low-income pensioners and low-income working population analysis	Middle-income working population and low-income working population analysis
**Cardiovascular agents**		
During-before the announcement	3.00*** (0.26)	0.20 (0.11)
After-during the announcement	-2.27*** (0.26)	0.33***(0.11)
**Anti-hyperlipidemics**		
During-before the announcement	1.20***(0.11)	0.10 (0.06)
After-during the announcement	-0.05 (0.11)	0.03 (0.06)
**Endocrine/metabolic agents**		
During-before the announcement	2.54***(0.20)	0.01 (0.08)
After-during the announcement	-1.31***(0.18)	0.07 (0.07)
**Central nervous system agents**		
During-before the announcement	2.41*** (0.20)	-0.01 (0.09)
After-during the announcement	1.01*** (0.22)	0.16 (0.12)
**Diabetes drugs**		
During-before the announcement	0.92*** (0.15)	-0.05 (0.08)
After-during the announcement	0.09 (0.11)	0.10 (0.05)
**Dermatological**		
During-before the announcement	0.40***(0.16)	0.12 (0.09)
After-during the announcement	-0.69***(0.14)	0.12** (0.06)
**Gastrointestinal drugs**		
During-before the announcement	0.10***(0.04)	0.00 (0.02)
After-during the announcement	-0.01 (0.03)	0.02 (0.01)
**Analgesics/anti-inflammatories**		
During-before the announcement	1.02***(0.12)	0.04 (0.06)
After-during the announcement	-0.86***(0.11)	0.00 (0.05)
**Eye, Ear, nose and throat preparations**		
During-before the announcement	0.85***(0.12)	-0.02 (0.03)
After-during the announcement	-1.88*** (0.12)	-0.04 (0.03)
**Anti-infective**		
During-before the announcement	0.02 (0.06)	0.01 (0.04)
After-during the announcement	0.24*** (0.04)	0.06** (0.03)
**Upper respiratory agents**		
During-before the announcement	0.12*** (0.04)	0.08** (0.04)
After-during the announcement	-0.05 (0.04)	0.05 (0.04)
**Genitourinary agents**		
During-before the announcement	0.17*** (0.07)	-0.09** (0.05)
After-during the announcement	-0.20*** (0.07)	-0.08 (0.05)
**Pulmonary drugs**		
During-before the announcement	1.24***(0.18)	0.12 (0.08)
After-during the announcement	-0.53*** (0.13)	0.07 (0.06)

The table contains Difference-in-Differences estimates from linear regression models with robust standard errors. Each cell contains results of the model from different therapeutic groups. All regressions include age and age2, and time dummies. Within each cell, we first report the estimated coefficients; we then report in parentheses robust standard errors. The therapeutic groups are sorted by price-elasticity (at the top the most inelastic, while at the bottom the most elastic).

Significance levels

***p < 0.01

**p < 0.05.

In order to fully analyse the effect of the reform, we need to compare the behaviour of individuals after the reform’s announcement with their behaviour after its implementation. Accordingly, the general analysis reflected an increase (16.60) in consumption among low-income pensioners, which fully compensated for the subsequent reduction (-13.94) (cf. Table 4).

By therapeutic groups for low-income pensioners, we observed that those therapeutic groups that fully compensated for the reduction with a previous increase were cardiovascular agents (with a decrease of 2.27 after the reform preceded by an increase of 3.00, the total change was of 0.73); endocrine/metabolic agents (with a post-reform decrease of 1.31 and a pre-reform increase of 2.54 the total change was 1.23); analgesics/anti-inflammatories (with a post-reform decrease of 0.86 and a pre-reform increase of 1.02 the total change was of 0.16); and pulmonary drugs (with a decrease of 0.53 after the reform preceded by an increase of 1.24 the total change was 0.71). By contrast, those therapeutic groups that only partially compensated the reduction experimented after the reform’s implementation were dermatological (with a decrease of 0.69 after the reform preceded by an increase of 0.40 the total change was -0.29); eye, ear, nose and throat preparations (with a post-reform decrease of 1.88 and pre-reform increase of 0.85 the total change was -1.03); and, genitourinary agents (with a post-reform decrease of 0.20 and a pre-reform increase of 0.17 the total change was -0.03). In addition, central nervous system agents experienced increased consumption after the reform’s announcement (2.41), but also increased after its implementation. Furthermore, anti-hyperlipidemics (1.20), diabetes drugs (0.92) gastrointestinal drugs (0.10), upper respiratory agents (0.12) showed increased consumption after the reform’s announcement, but they were not followed by statistically significant results after the reform’s implementation. For middle-income working population, there were no statistically significant results to highlight (cf. Table 5).

**Fig 2:**
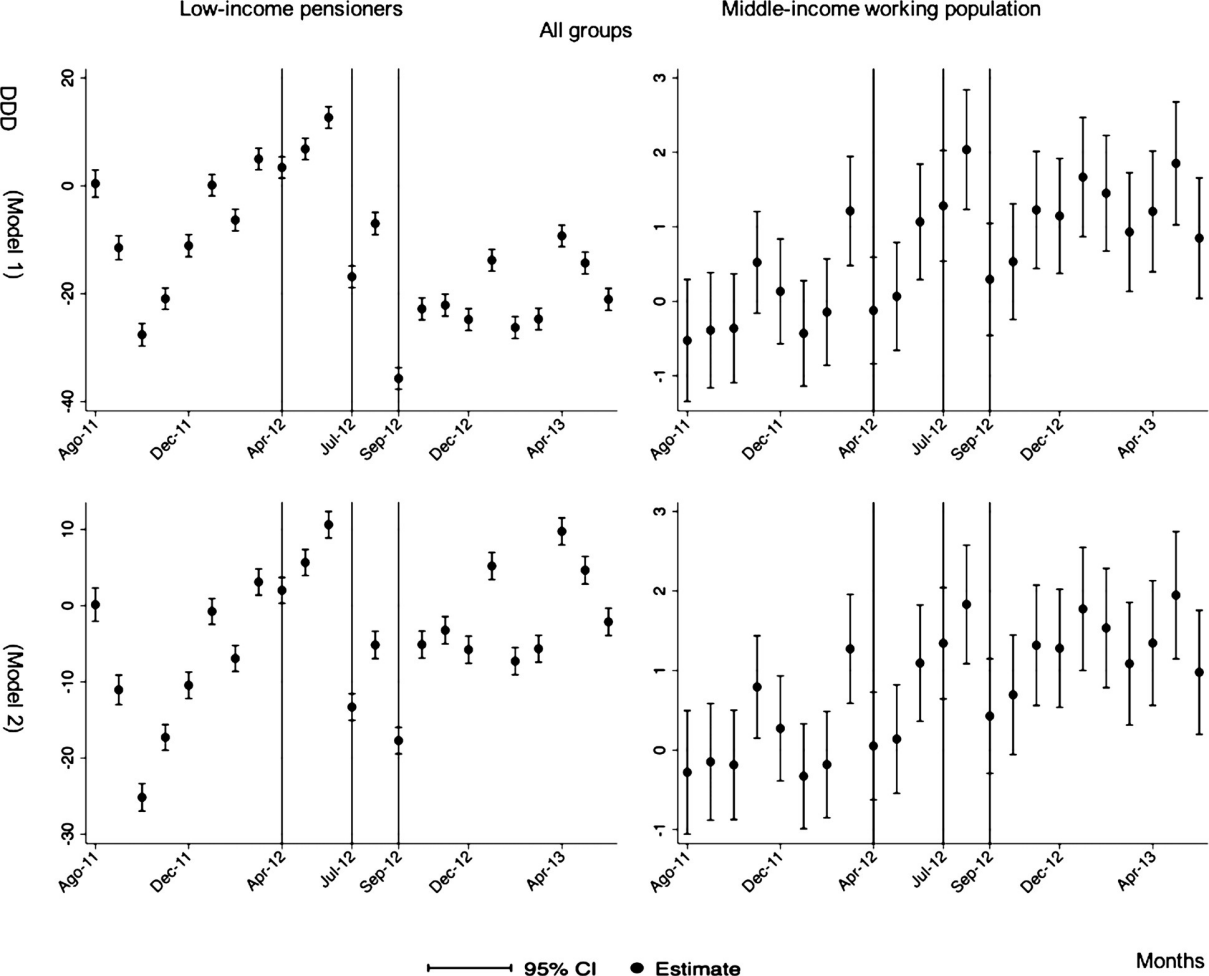
Monthly follow-up effect of the cost-sharing change on the pharmaceutical consumption. The graphics contain Difference-in-Differences estimates from linear regression models with robust standard errors with a 95% confidence interval (CI). All regressions include age and age2, and time dummies. We used for the analysis bar graphics with standard errors. The monthly period covers from August 2011 to June 2013; April 2012: April 20th, 2012 was the date on which the reform was announced; July 2012: July 1st, 2012 was the date on which the reform entered into force (date of cost-sharing change); September 2012: September 1st, 2012 was the date on which 426 were excluded from public provision. We provide two models: Model 1 refers to the regression model run with all the medicines of the database (including the drugs excluded from public provision). Model 2 refers to the regression model run without the drugs excluded from public provision.

The monthly follow-up for low-income pensioners in the general analysis did not reflect a clear recovery in the number of monthly DDDs per individual. The estimations showed the highest reduction (-35.74) in September 2012 followed by a small recovery in November 2012 (i.e., changing from -35.74 in September to -22.13 in November 2012). Notwithstanding this recovery, we did not observe that the consumption level that would have existed in absence of the reform was reached in the following months. For middle-income working population, there were only statistically significant results in August 2012, January 2013 and May 2013 (with increases of 2.04, 1.67 and 1.85 in the number of monthly DDDs per individual). However, the results of the monthly analysis without the list of 426 drugs excluded from public provision showed a slight recovery in consumption (cf. Fig 2 and S1–S3 Tables).

**Fig 3:**
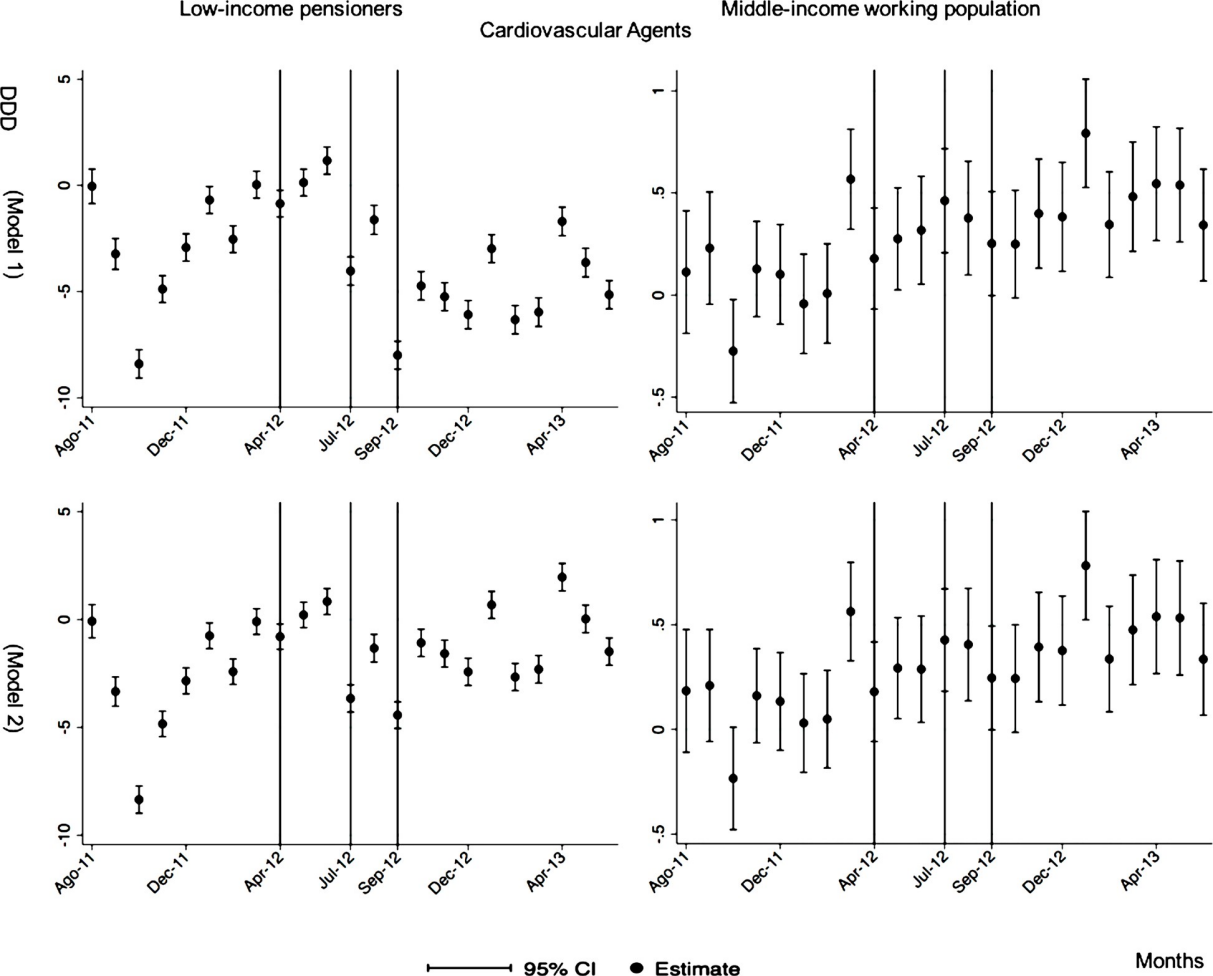
Monthly follow-up effect of the cost-sharing change on the pharmaceutical consumption: Cardiovascular Agents. The figures contain Difference-in-Difference estimates from linear regression models with robust standard errors with a 95% confidence interval (CI). All regressions include age and age2, and time dummies. We used for the analysis bar graphics with standard errors. The monthly period covers from August 2011 to June 2013; April 2012: April 20th, 2012 was the date on which the reform was announced; July 2012: July 1st, 2012 was the date on which the reform entered into force (date of cost-sharing change); September 2012: September 1st, 2012 was the date on which 426 were excluded from public provision. Model 1 refers to the regression model run with all the medicines of the database (including the drugs excluded from public provision). Model 2 refers to the regression model run without the drugs excluded from public provision.

**Fig 4:**
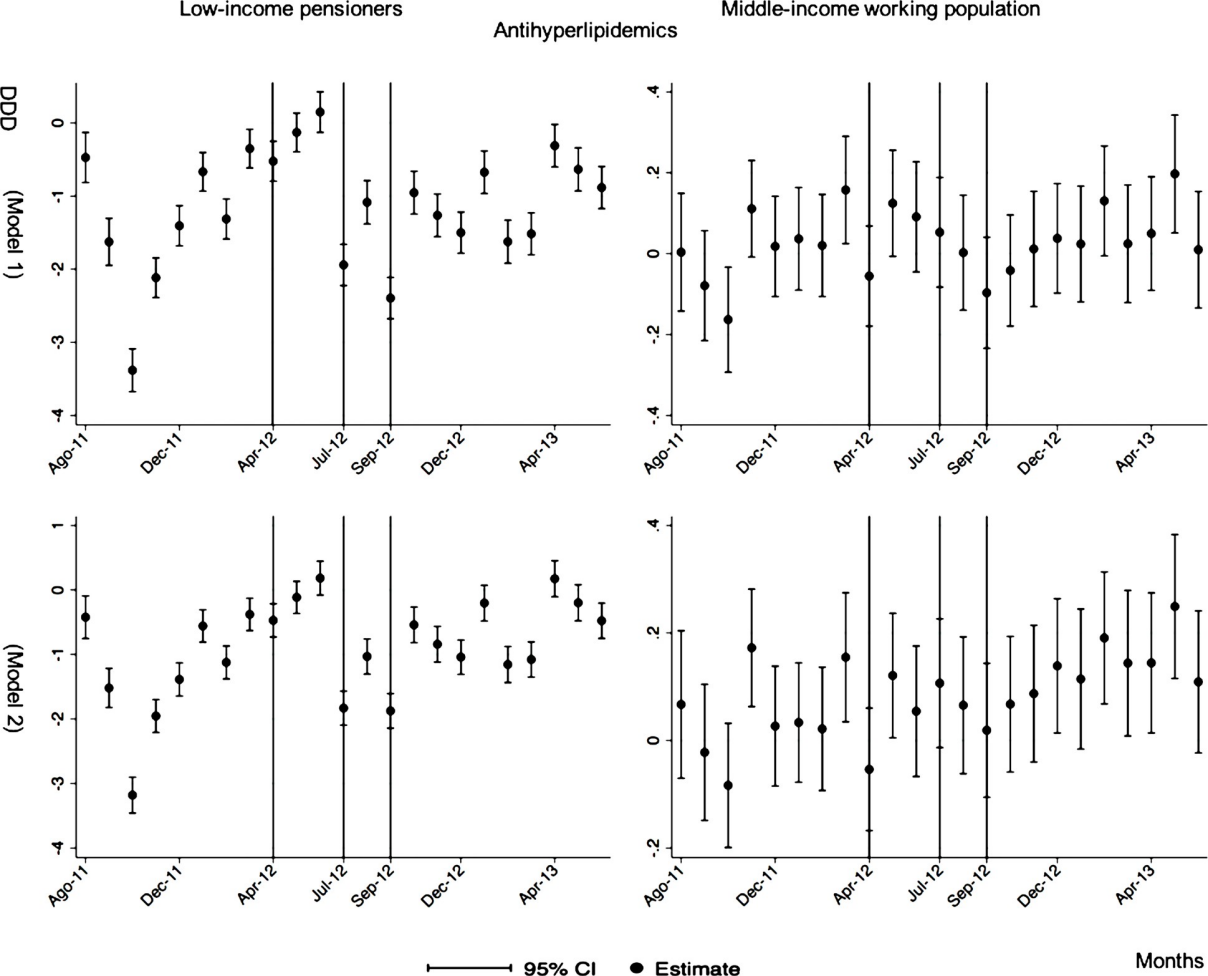
Monthly follow-up effect of the cost-sharing change on the pharmaceutical consumption: Antihyperlipidemics. The figures contain Difference-in-Difference estimates from linear regression models with robust standard errors with a 95% confidence interval (CI). All regressions include age and age2, and time dummies. We used for the analysis bar graphics with standard errors. The monthly period covers from August 2011 to June 2013; April 2012: April 20th, 2012 was the date on which the reform was announced; July 2012: July 1st, 2012 was the date on which the reform entered into force (date of cost-sharing change); September 2012: September 1st, 2012 was the date on which 426 were excluded from public provision. Model 1 refers to the regression model run with all the medicines of the database (including the drugs excluded from public provision). Model 2 refers to the regression model run without the drugs excluded from public provision.

**Fig 5:**
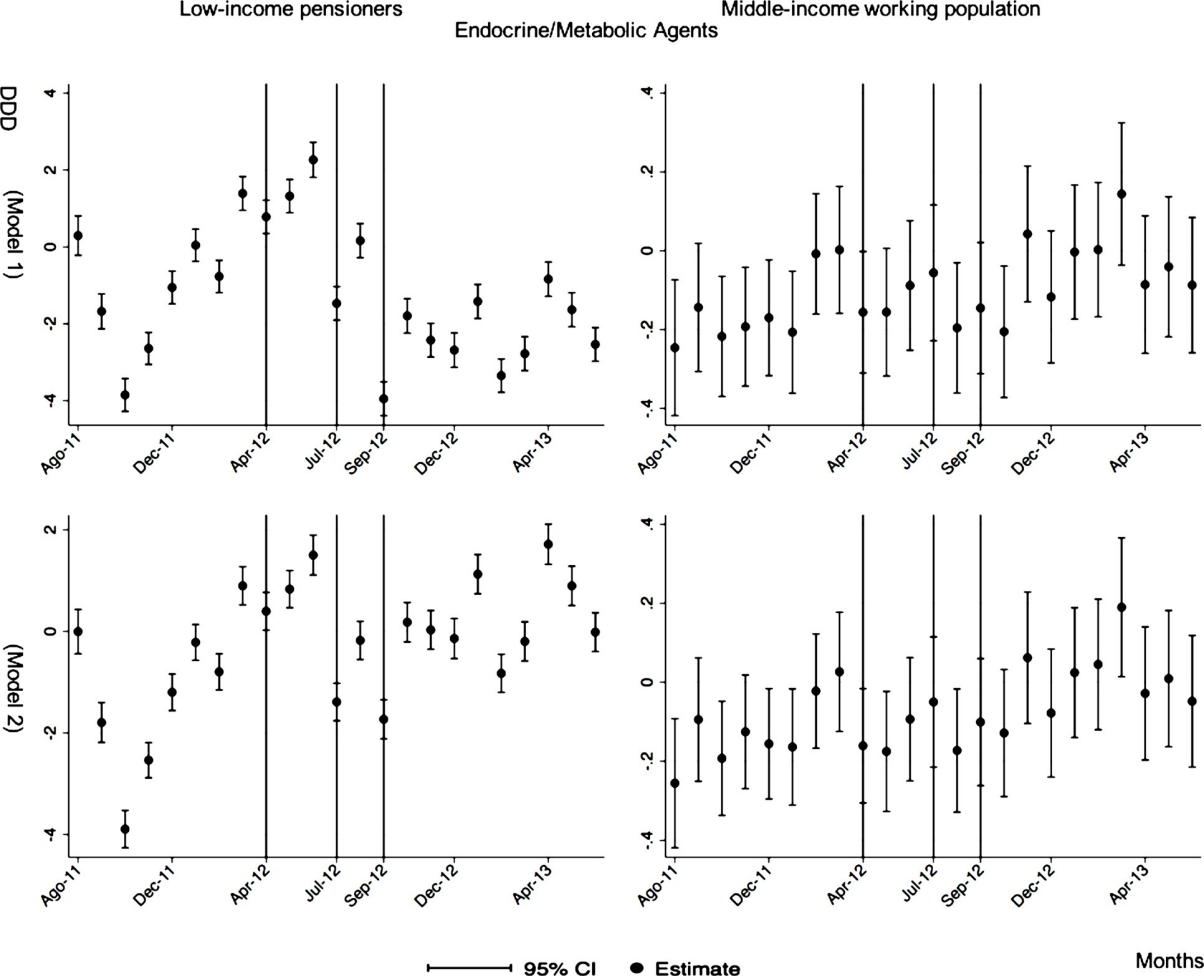
Monthly follow-up effect of the cost-sharing change on the pharmaceutical consumption: Endocrine/Metabolic Agents. The figures contain Difference-in-Difference estimates from linear regression models with robust standard errors with a 95% confidence interval (CI). All regressions include age and age2, and time dummies. We used for the analysis bar graphics with standard errors. The monthly period covers from August 2011 to June 2013; April 2012: April 20th, 2012 was the date on which the reform was announced; July 2012: July 1st, 2012 was the date on which the reform entered into force (date of cost-sharing change); September 2012: September 1st, 2012 was the date on which 426 were excluded from public provision. Model 1 refers to the regression model run with all the medicines of the database (including the drugs excluded from public provision). Model 2 refers to the regression model run without the drugs excluded from public provision.

**Fig 6:**
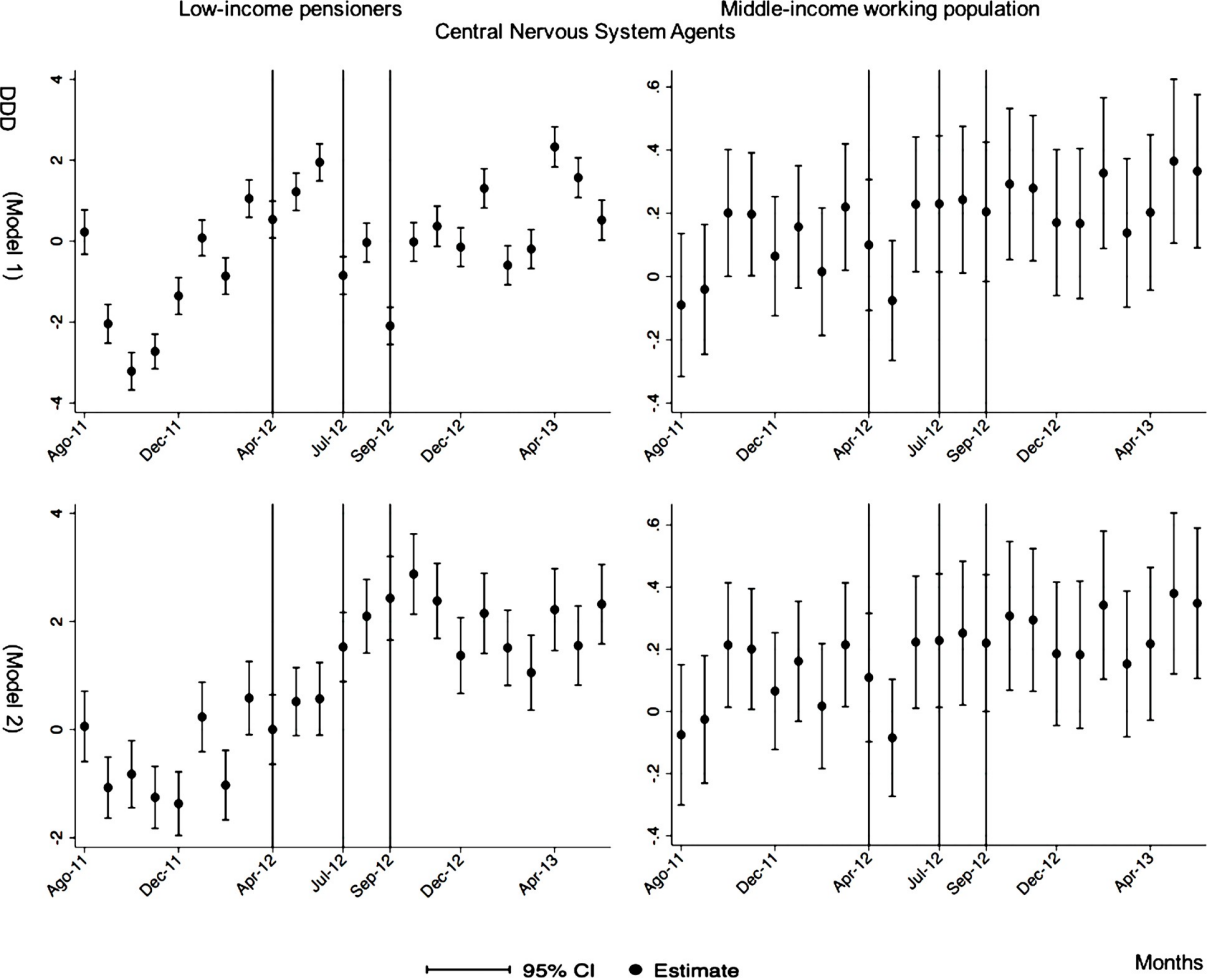
Monthly follow-up effect of the cost-sharing change on the pharmaceutical consumption: Central Nervous System Agents. The figures contain Difference-in-Difference estimates from linear regression models with robust standard errors with a 95% confidence interval (CI). All regressions include age and age2, and time dummies. We used for the analysis bar graphics with standard errors. The monthly period covers from August 2011 to June 2013; April 2012: April 20th, 2012 was the date on which the reform was announced; July 2012: July 1st, 2012 was the date on which the reform entered into force (date of cost-sharing change); September 2012: September 1st, 2012 was the date on which 426 were excluded from public provision. Model 1 refers to the regression model run with all the medicines of the database (including the drugs excluded from public provision). Model 2 refers to the regression model run without the drugs excluded from public provision.

**Fig 7:**
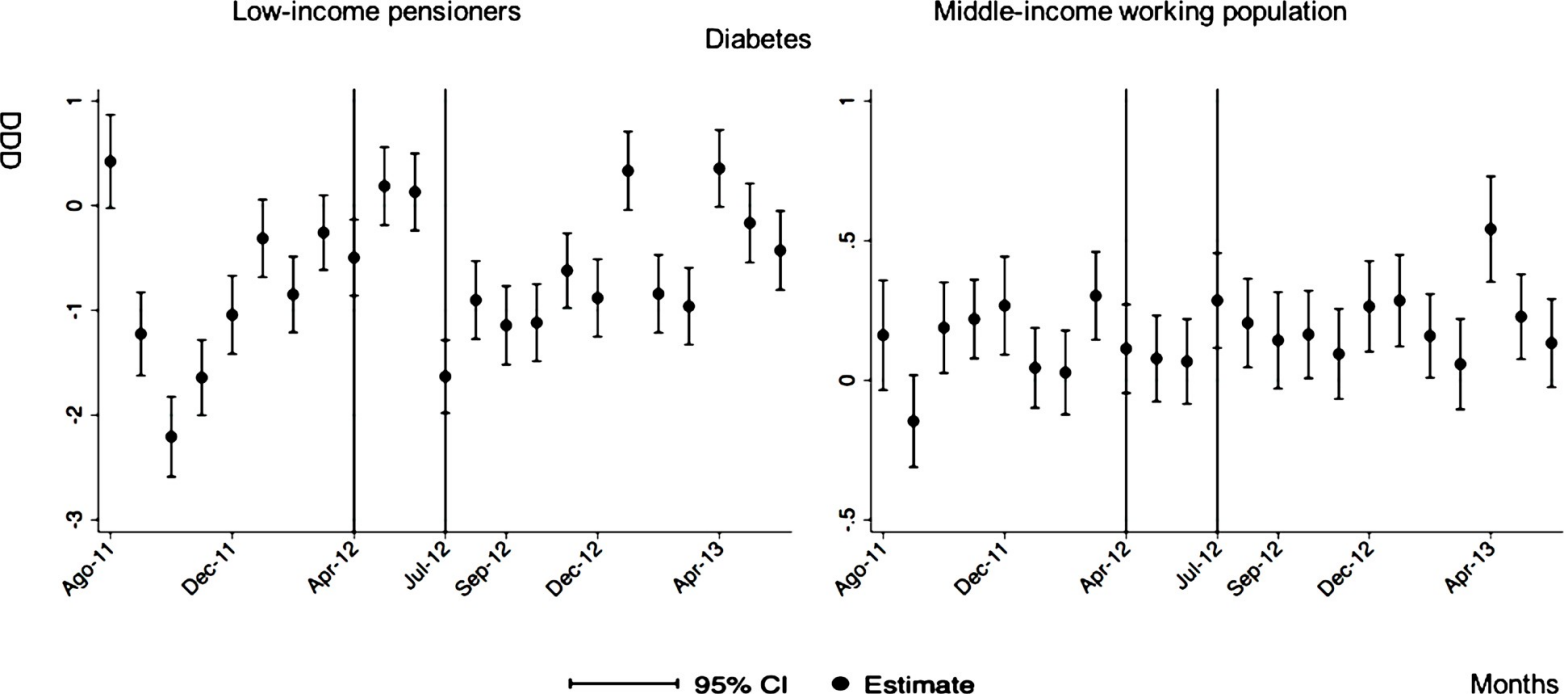
Monthly follow-up effect of the cost-sharing change on the pharmaceutical consumption: Diabetes. The figures contain Difference-in-Difference estimates from linear regression models with robust standard errors with a 95% confidence interval (CI). All regressions include age and age2, and time dummies. We used for the analysis bar graphics with standard errors. The monthly period covers from August 2011 to June 2013; April 2012: April 20th, 2012 was the date on which the reform was announced; July 2012: July 1st, 2012 was the date on which the reform entered into force (date of cost-sharing change). As this therapeutic group does not contain medicines from the list of 426 drugs excluded from public provision, we only provide one model.

**Fig 8:**
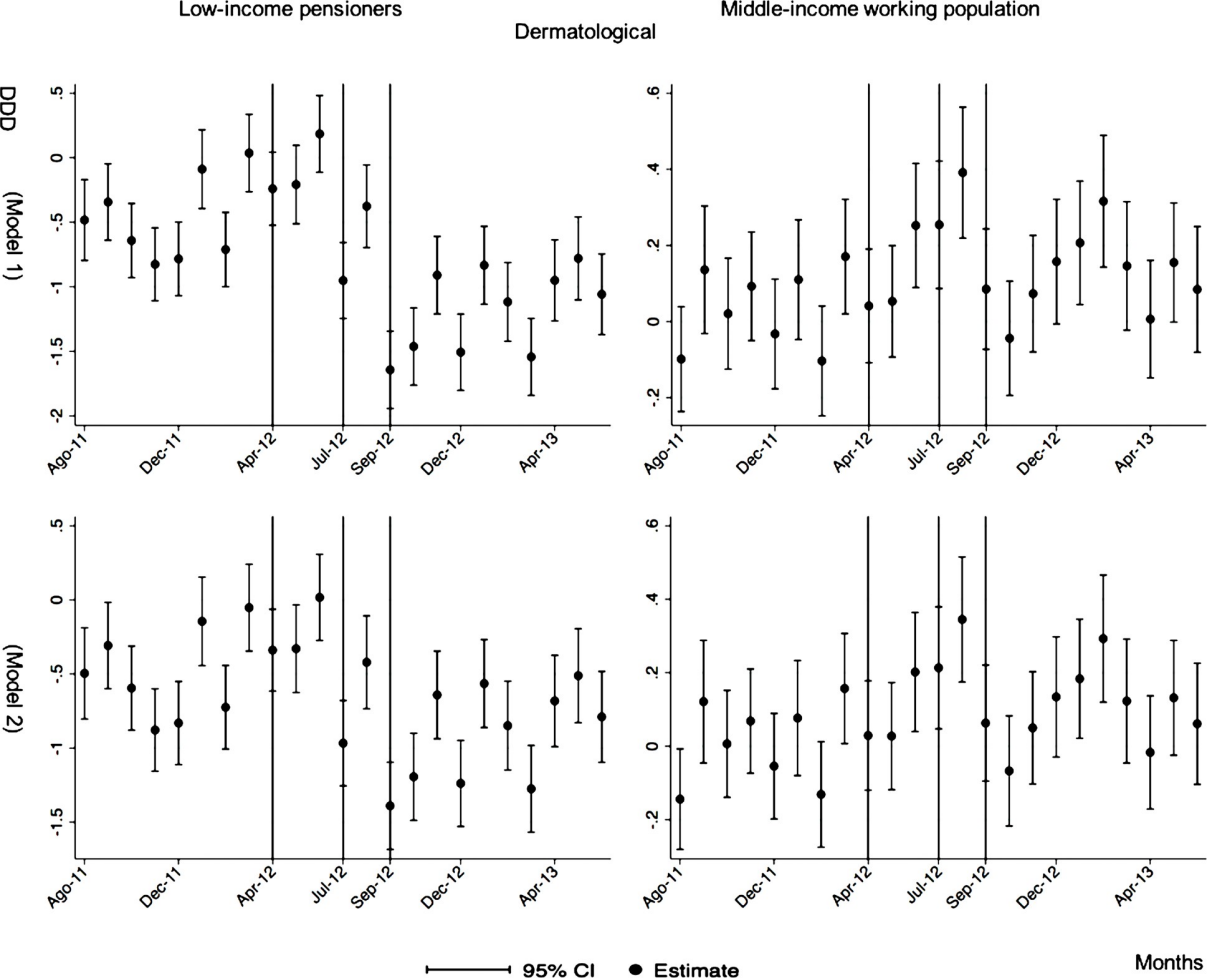
Monthly follow-up effect of the cost-sharing change on the pharmaceutical consumption: Dermatological. The figures contain Difference-in-Difference estimates from linear regression models with robust standard errors with a 95% confidence interval (CI). All regressions include age and age2, and time dummies. We used for the analysis bar graphics with standard errors. The monthly period covers from August 2011 to June 2013; April 2012: April 20th, 2012 was the date on which the reform was announced; July 2012: July 1st, 2012 was the date on which the reform entered into force (date of cost-sharing change); September 2012: September 1st, 2012 was the date on which 426 were excluded from public provision. Model 1 refers to the regression model run with all the medicines of the database (including the drugs excluded from public provision). Model 2 refers to the regression model run without the drugs excluded from public provision.

**Fig 9:**
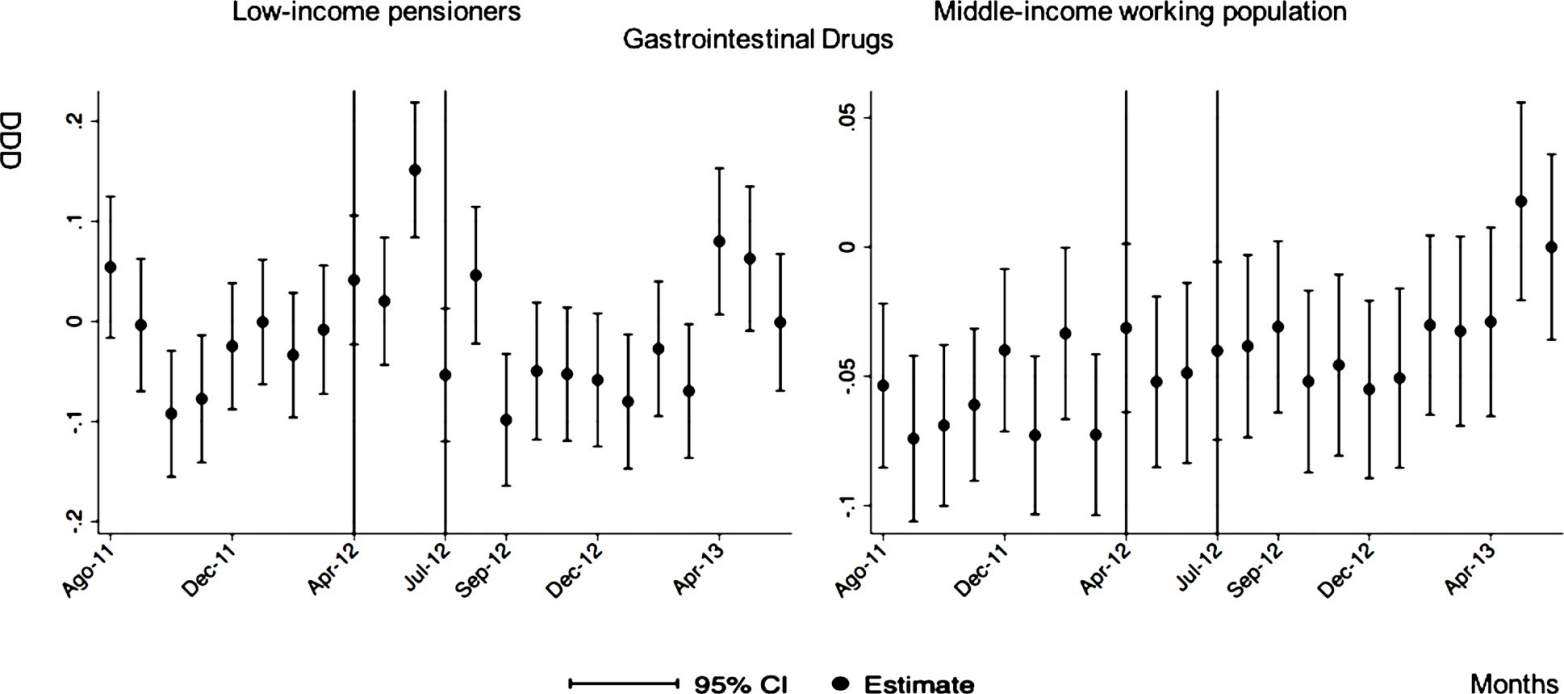
Monthly follow-up effect of the cost-sharing change on the pharmaceutical consumption: Gastrointestinal drugs. The figures contain Difference-in-Difference estimates from linear regression models with robust standard errors with a 95% confidence interval (CI). All regressions include age and age2, and time dummies. We used for the analysis bar graphics with standard errors. The monthly period covers from August 2011 to June 2013; April 2012: April 20th, 2012 was the date on which the reform was announced; July 2012: July 1st, 2012 was the date on which the reform entered into force (date of cost-sharing change). As this therapeutic group does not contain medicines from the list of 426 drugs excluded from public provision, we only provide one model.

**Fig 10:**
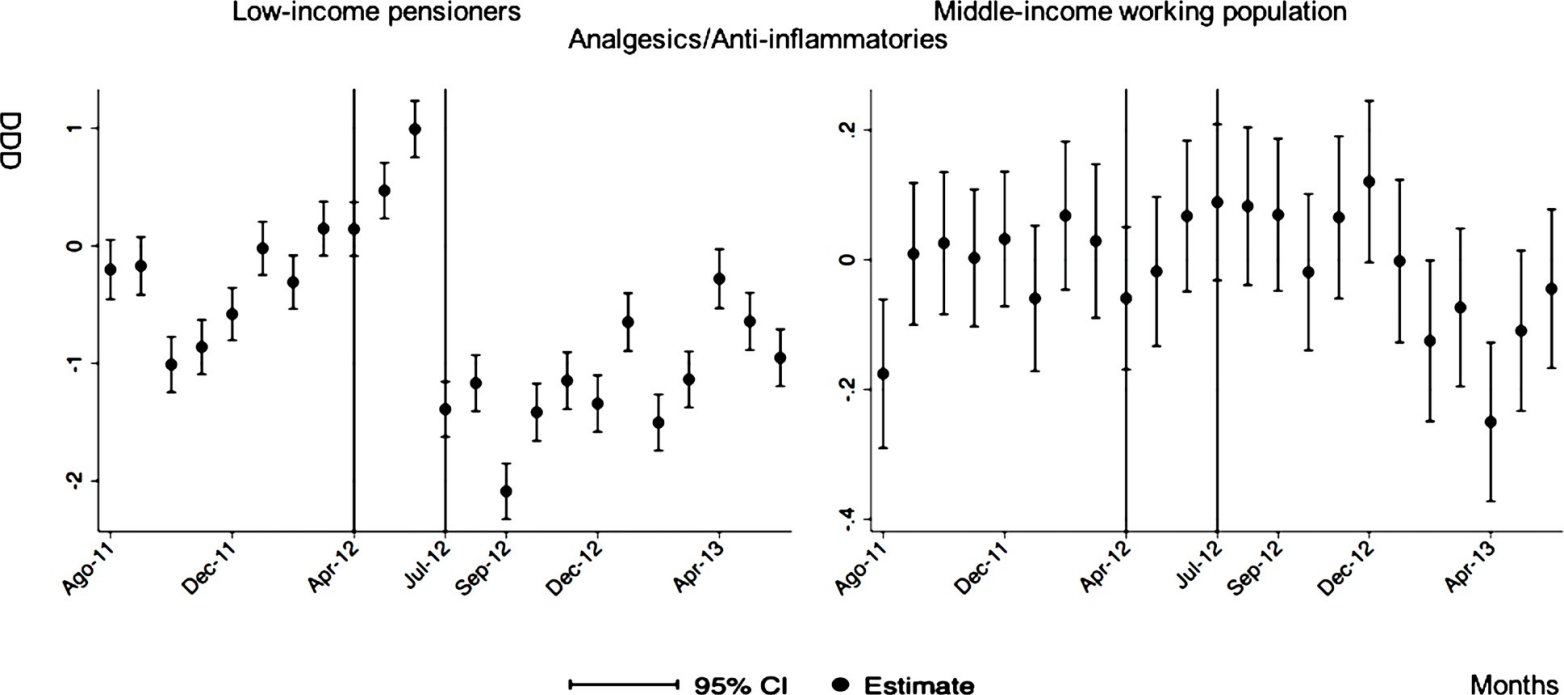
Monthly follow-up effect of the cost-sharing change on the pharmaceutical consumption: Analgesics/Anti-inflammatories. The figures contain Difference-in-Difference estimates from linear regression models with robust standard errors with a 95% confidence interval (CI). All regressions include age and age2, and time dummies. We used for the analysis bar graphics with standard errors. The monthly period covers from August 2011 to June 2013; April 2012: April 20th, 2012 was the date on which the reform was announced; July 2012: July 1st, 2012 was the date on which the reform entered into force (date of cost-sharing change). As this therapeutic group does not contain medicines from the list of 426 drugs excluded from public provision, we only provide one model.

**Fig 11:**
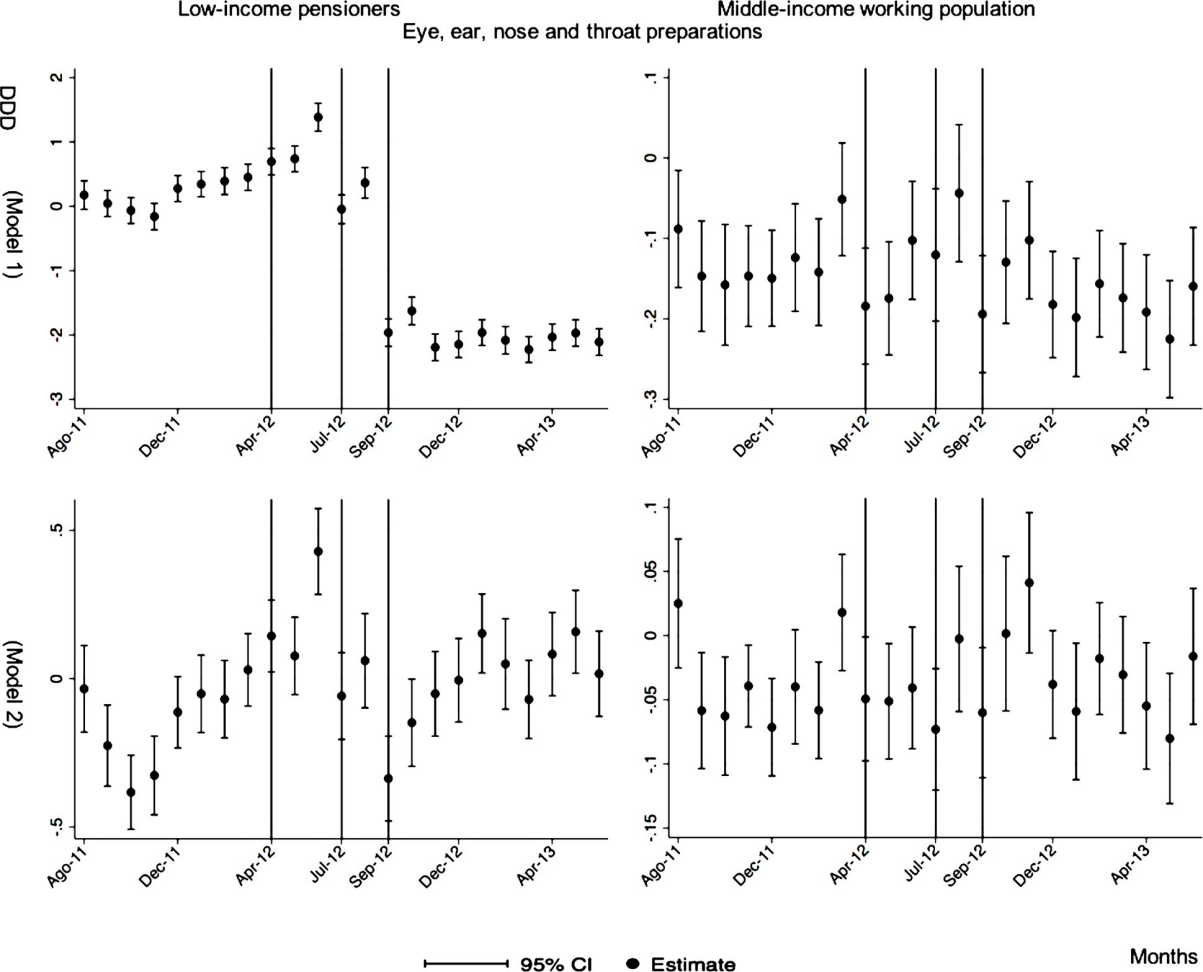
Monthly follow-up effect of the cost-sharing change on the pharmaceutical consumption: Eye, ear, nose and throat preparations. The figures contain Difference-in-Difference estimates from linear regression models with robust standard errors with a 95% confidence interval (CI). All regressions include age and age2, and time dummies. We used for the analysis bar graphics with standard errors. The monthly period covers from August 2011 to June 2013; April 2012: April 20th, 2012 was the date on which the reform was announced; July 2012: July 1st, 2012 was the date on which the reform entered into force (date of cost-sharing change); September 2012: September 1st, 2012 was the date on which 426 were excluded from public provision. Model 1 refers to the regression model run with all the medicines of the database (including the drugs excluded from public provision). Model 2 refers to the regression model run without the drugs excluded from public provision.

**Fig 12:**
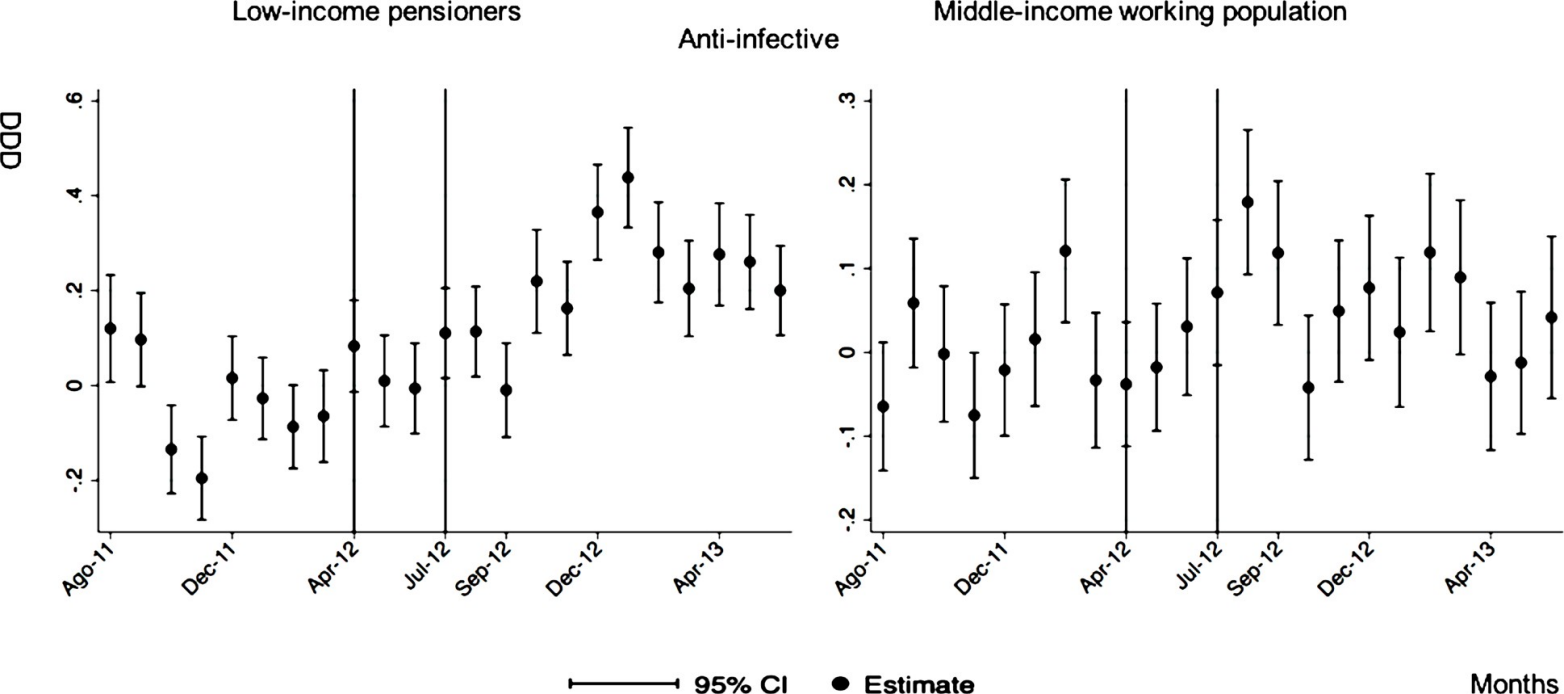
Monthly follow-up effect of the cost-sharing change on the pharmaceutical consumption: Anti-infective. The figures contain Difference-in-Difference estimates from linear regression models with robust standard errors with a 95% confidence interval (CI). All regressions include age and age2, and time dummies. We used for the analysis bar graphics with standard errors. The monthly period covers from August 2011 to June 2013; April 2012: April 20th, 2012 was the date on which the reform was announced; July 2012: July 1st, 2012 was the date on which the reform entered into force (date of cost-sharing change). As this therapeutic group does not contain medicines from the list of 426 drugs excluded from public provision, we only provide one model.

**Fig 13:**
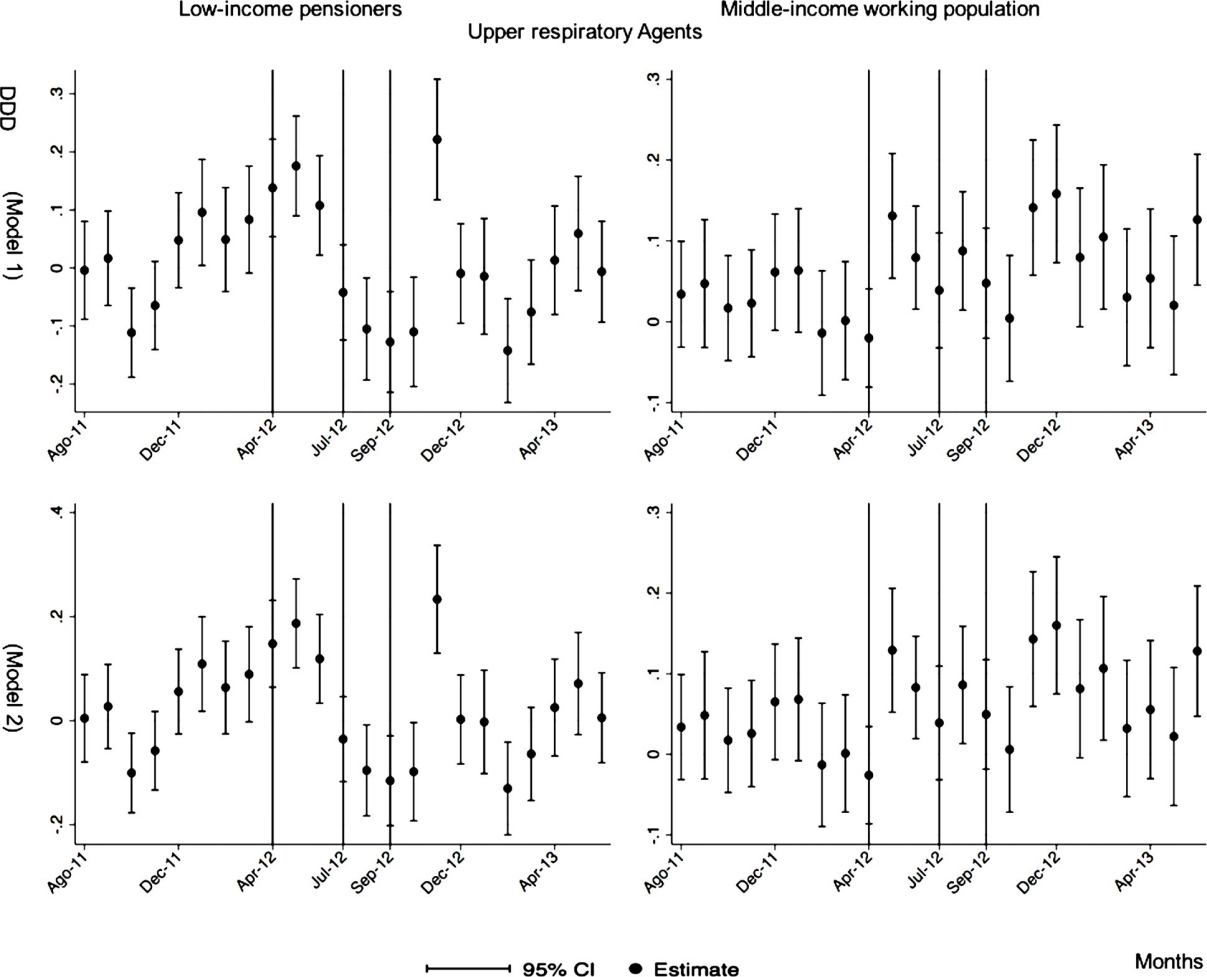
Monthly follow-up effect of the cost-sharing change on the pharmaceutical consumption: Upper Respiratory Agents. The figures contain Difference-in-Difference estimates from linear regression models with robust standard errors with a 95% confidence interval (CI). All regressions include age and age2, and time dummies. We used for the analysis bar graphics with standard errors. The monthly period covers from August 2011 to June 2013; April 2012: April 20th, 2012 was the date on which the reform was announced; July 2012: July 1st, 2012 was the date on which the reform entered into force (date of cost-sharing change); September 2012: September 1st, 2012 was the date on which 426 were excluded from public provision. Model 1 refers to the regression model run with all the medicines of the database (including the drugs excluded from public provision). Model 2 refers to the regression model run without the drugs excluded from public provision.

**Fig 14:**
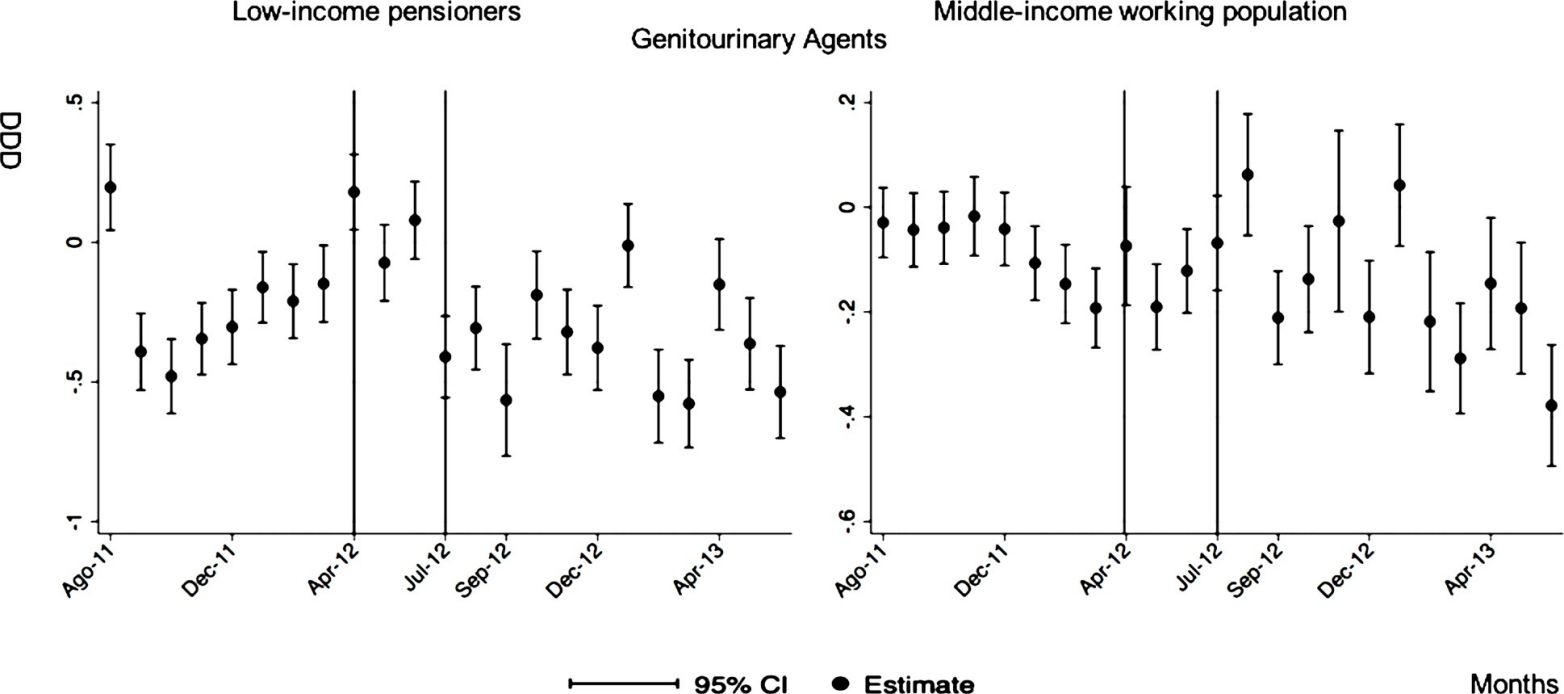
Monthly follow-up effect of the cost-sharing change on the pharmaceutical consumption: Genitourinary Agents. The figures contain Difference-in-Difference estimates from linear regression models with robust standard errors with a 95% confidence interval (CI). All regressions include age and age2, and time dummies. We used for the analysis bar graphics with standard errors. The monthly period covers from August 2011 to June 2013; April 2012: April 20th, 2012 was the date on which the reform was announced; July 2012: July 1st, 2012 was the date on which the reform entered into force (date of cost-sharing change). As this therapeutic group does not contain medicines from the list of 426 drugs excluded from public provision, we only provide one model.

**Fig 15:**
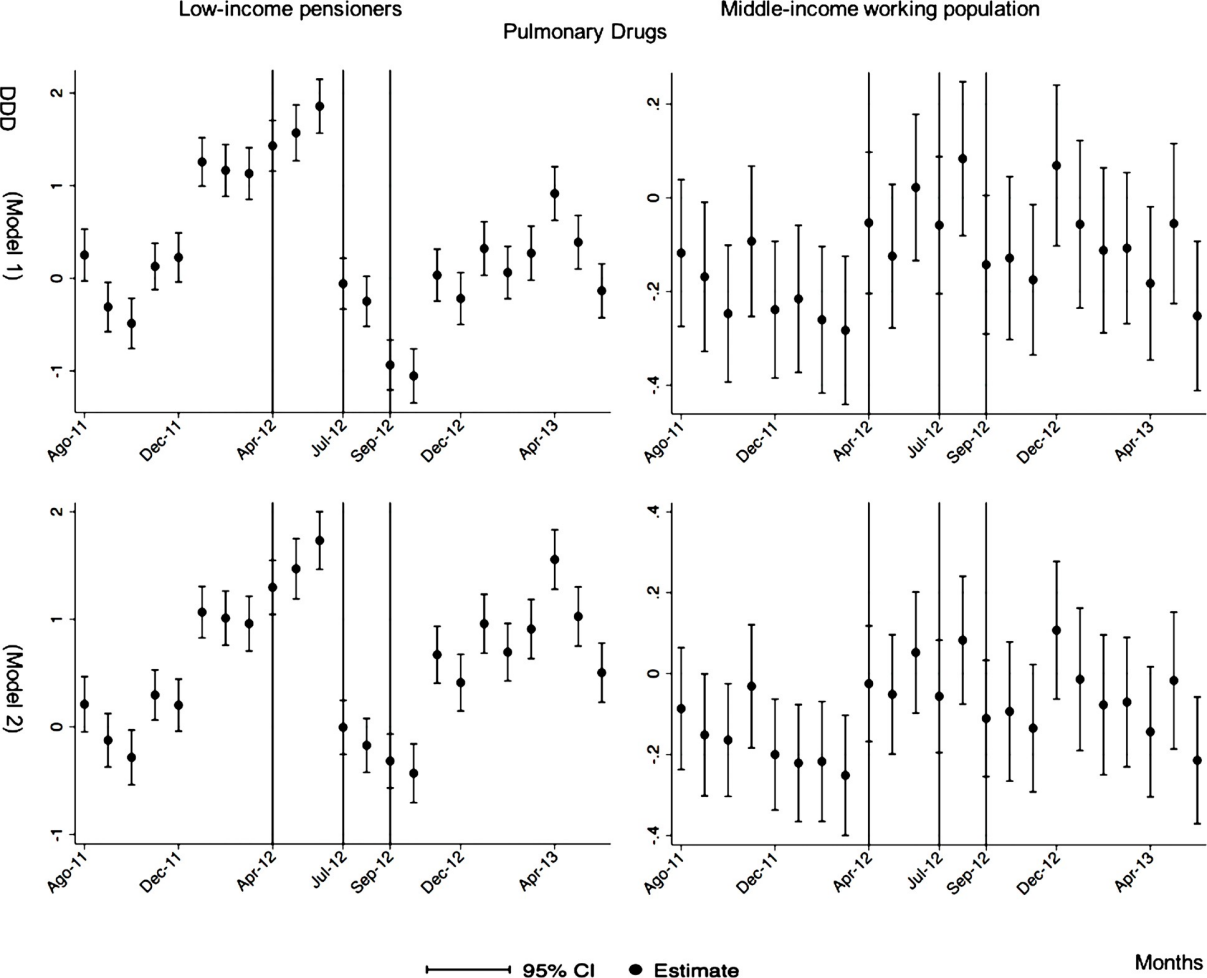
Monthly follow-up effect of the cost-sharing change on the pharmaceutical consumption: Pulmonary Drugs. The figures contain Difference-in-Difference estimates from linear regression models with robust standard errors with a 95% confidence interval (CI). All regressions include age and age2, and time dummies. We used for the analysis bar graphics with standard errors. The monthly period covers from August 2011 to June 2013; April 2012: April 20th, 2012 was the date on which the reform was announced; July 2012: July 1st, 2012 was the date on which the reform entered into force (date of cost-sharing change); September 2012: September 1st, 2012 was the date on which 426 were excluded from public provision. Model 1 refers to the regression model run with all the medicines of the database (including the drugs excluded from public provision). Model 2 refers to the regression model run without the drugs excluded from public provision.

The negative effect in the overall analysis for almost all the therapeutic groups for low-income pensioners (cardiovascular agents’ diseases, eye, ear, nose and throat preparations, endocrine/metabolic agents, analgesics/anti-inflammatories, dermatological, pulmonary drugs and genitourinary agents) was maintained in the months following the reform. The only therapeutic group that seemed to recover its consumption level was pulmonary drugs. Unlike the overall analysis, the monthly follow-up study provided statistically significant decreases for anti-hyperlipidemics and diabetes drugs in some months without a clear recovery of the consumption level. Regarding upper respiratory agents, we detected an increase in November 2012. However, for most of the therapeutic groups, we observed the biggest reduction in September 2012, which was followed by a minor recovery between October and November 2012 that did not hold in the following months. We also found that most of the therapeutic groups that included a significant percentage of drugs from the list of excluded medicines from public provision (i.e., anti-hyperlipidemics; cardiovascular agents; dermatological; endocrine/metabolic agents; eye, ear, nose and throat preparations; pulmonary drugs) were also those that notably reduced consumption over months. Accordingly, the therapeutic groups that contained excluded drugs reported a minor negative coefficient or even a recovery in consumption when we ran the regression model without the excluded drugs (cf. Figs 3–15 and S1, S3 and S5 Tables). For middle-income working population, there were some months in which eye, ear, nose and throat preparations and analgesics/anti-inflammatories reflected a small decrease in their number of monthly DDDs per individual. Apart from this, the results for the remaining therapeutic groups that provided statistically significant estimates (cardiovascular agents, diabetes drugs, dermatological and anti-infective) showed a positive effect of the cost sharing (cf. Figs 3–15 and S2 and S3 Tables).

Additionally, regarding those individuals with comorbidities (estimated as people who have been dispensed medicines from two or more therapeutic groups), we observed a statistically significant decrease in consumption (-20.37) among low-income pensioners, which was preceded by a previous increase of 5.56. By contrast, we detected an increase in consumption (2.60) after the reform, among patients who were dispensed medicines from one-therapeutic group. For middle-income working population, we observed a statistically significant increase in consumption (0.43) after the reform among patients who were dispensed medicines from one therapeutic group (cf. Table 6).

**Table 6:** Overall effect of the cost-sharing change on the pharmaceutical consumption by comorbidities ^NOTE^.

	Low-income pensioners and low-income working population analysis	Middle-income working population and low-income working population analysis
**People who have been dispensed medicines of two or more therapeutic groups**		
During-before the announcement	5.56***(1.12)	0.56 (1.44)
After-during the announcement	-20.37***(0.98)	-1.27 (1.16)
**People who have been dispensed medicines of one therapeutic group**		
During-before the announcement	1.15***(0.35)	0.01 (0.16)
After-during the announcement	2.60*** (0.32)	0.43*** (0.14)

The table contains Difference-in-Difference estimates from linear regression models with robust standard errors. Each cell contains results of the model from different therapeutic groups. All regressions include age and age2, and time dummies. Within each cell, we first report the estimated coefficients; we then report in parentheses robust standard errors. The therapeutic groups are sorted by price-elasticity (at the top the most inelastic, while at the bottom the most elastic).

NOTE: We were not able to exactly know if the individuals of the sample had comorbidities because we did not have information about the specific diseases of each individual. Therefore, we have approached the case of a patient having comorbidities as one who has been dispensed medicines of two or more therapeutic groups. We compared these results with the effect of a patient who has been dispensed medicines from one therapeutic group.

Significance levels

***p < 0.01

**p < 0.05.

**Fig 16:**
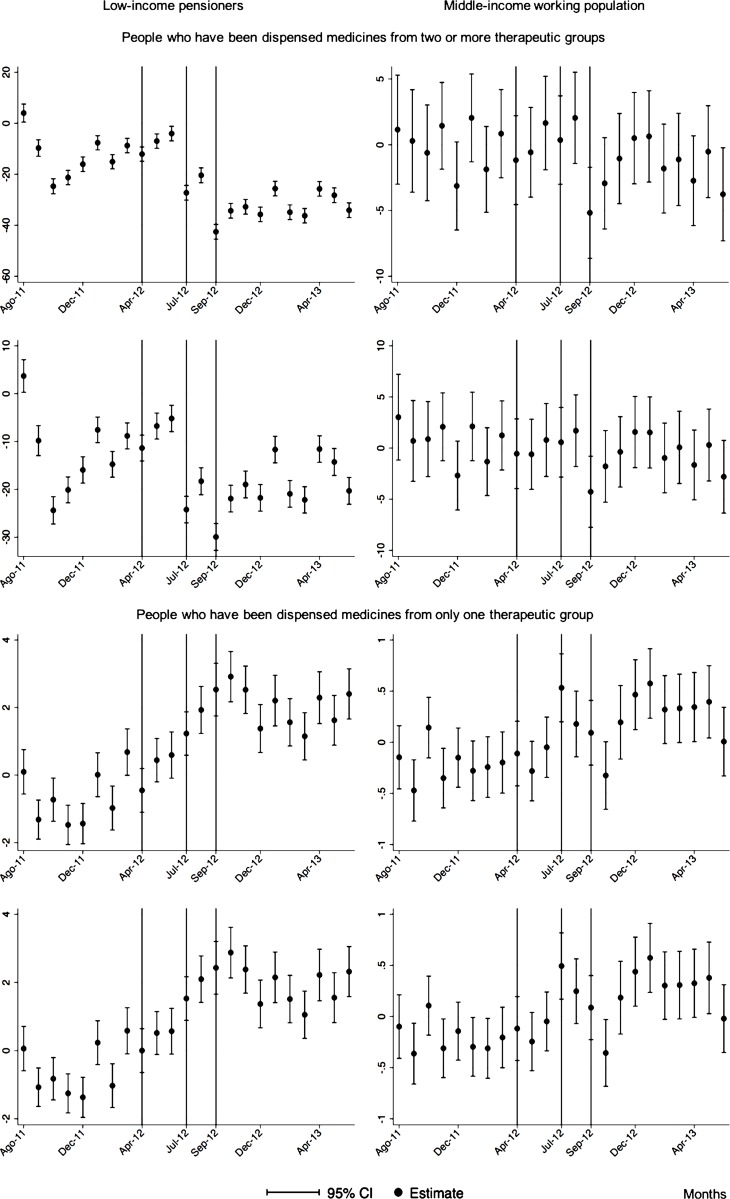
Monthly follow-up effect of the cost-sharing change on the pharmaceutical consumption by comorbidities ^NOTE^. The graphics contain Difference-in-Differences estimates from linear regression models with robust standard errors with a 95% confidence interval (CI). All regressions include age and age2, and time dummies. We used for the analysis bar graphics with standard errors. The monthly period covers from August 2011 to June 2013; April 2012: April 20th, 2012 was the date on which the reform was announced; July 2012: July 1st, 2012 was the date on which the reform entered into force (date of cost-sharing change); September 2012: September 1st, 2012 was the date on which 426 were excluded from public provision. Model 1 refers to the regression model run with all the medicines of the database (including the drugs excluded from public provision). Model 2 refers to the regression model run without the drugs excluded from public provision. NOTE: We were not able to exactly know if the individuals of the sample had comorbidities because we did not have information about the specific diseases of each individual. Therefore, we have approached the case of a patient having comorbidities as one who has been dispensed medicines from two or more therapeutic groups. We compared these results with the effect of a patient who was dispensed medicines from one therapeutic group.

Although during the months after the reform we did not detect a consumption recovery among low-income pensioners with comorbidities, the consumption slightly recovered when we developed the analysis without the list of 426 medicines excluded from public provision. For middle-income working population, the estimates were not statistically significant. In the case of patients (low-income. Pensioners and middle-income working population) who were dispensed medicines from one therapeutic group, we observe an increasing trend over the months (cf. Fig 16 and S4 Table).

## 4. Discussion

There was a negative impact of the cost-sharing change on the drug consumption of low-income pensioners, as was expected [9,10]. However, as the impact of the reform on pharmaceutical consumption was small in all therapeutic groups (cf. variation rates comparison in Table 3), individuals did not seem to be very sensitive to changes in drug prices, that is to say, the price elasticity of drugs is quite low [2,3,4,5,18,19]. This means that the reform had a ‘tax collection’ profile more than a ‘moral hazard’ reduction profile. Moreover, middle-income working population maintained its increasing consumption trend. This behaviour reflects that, as the literature maintains [9,10], middle-income individuals are not affected by co-payment increases.

Our analysis also shows the heterogeneity that exists of the cost-sharing effect by therapeutic groups with more marked reductions among the most inelastic therapeutic groups, which are those that mainly contain medicines for the treatment of chronic diseases. In addition, as the analysis of co-payment effects on consumption would be biased if existence of stockpiling were not considered, in this paper, we have highlighted the important role of stockpiling thus overcoming the limitations of previous studies that have analysed this reform. Despite some of these studies mentioning the existence of stockpiling [12,13,16], only [16] analysed it in depth. Unlike [12,13] studies, ours not only provides key information about the behaviour of individuals after a co-payment announcement, but also this key information was disaggregated by therapeutic groups. Specifically, we only detected this anticipation phenomenon (in the form of an increase in the number of monthly DDDs per individual after the reform’s announcement and its subsequent decrease after the reform’s implementation) among low-income pensioners. Stockpiling was especially marked, among the most inelastic groups, which are those related to chronic diseases [4,16] whose future need, as some studies have asserted [12,18,19], is predictable. As people with chronic illnesses were encouraged to stockpile [18,19], the increase detected after the reform’s announcement seemed to be a strategic behaviour of drug anticipation without loss of medication adherence risk (provided that the previous increase fully compensates the subsequent reduction).

Taking the above into account, cardiovascular agents’ diseases, eye, ear, nose and throat preparations, endocrine/metabolic agents, analgesics/anti-inflammatories, dermatological, pulmonary drugs and genitourinary agents reflected the negative effect of the cost-sharing change among low-income pensioners in the period immediately after the implementation of RDL 16/2012. This effect was compensated by previous stockpiling, except for eye, ear, nose and throat preparations, dermatological and genitourinary, where the negative coefficients after the implementation were greater than the previous positive coefficients. As the reduction observed in consumption within these therapeutic groups could not be totally attributed to previous stockpiling, we detected a risk of loss of medication adherence. By contrast, central nervous system and anti-infective drug groups did not appear to be affected by the cost-sharing change because these groups showed an increase in the number of monthly DDDs per individual after the reform. However, we cannot rule out that these consumption increases may be the result of an increase in the health needs among low-income pensioners with these health conditions, but with the information available, we were not able to test for this. For anti-hyperlipidemics, diabetes drugs, gastrointestinal drugs and upper respiratory agents, there were statistically significant increases after the reform’s announcement but none of them reflected statistically significant reductions after its implementation. Consequently, we were not able to verify with the overall analysis whether these groups were affected by the cost-sharing change or were subject to stockpiling practices.

For middle-income working population, there was an increase in pharmaceutical consumption among cardiovascular agents, dermatological and anti-infective drugs. These results together with those obtained in the descriptive statistics, would mean, as the literature holds [9,10], that co-payments affect vulnerable groups the most. Therefore, the middle-income working population is less sensitive to co-payment increases of ten percentage points (passing from a cost sharing percentage of 40% before the reform to ones of 50% after it). According to the descriptive statistics (cf. Fig 1), middle-income working population maintained (during the months after the reform) the increasing trend existing before the reform in almost all the therapeutic groups analysed.

The monthly study allowed us to verify better whether the reduction produced after July 1^st^, 2012 among low-income pensioners (the monthly follow-up analysis for middle-income working population did not provide any relevant information to highlight), was either a consequence of the previous stockpiling effect or a real loss in medication adherence. In both cases, for chronic and for acute therapeutic groups, there was not even a glimmer of a recovery in the pharmaceutical consumption existing before the reform. The fact that September 2012 showed (for the majority of therapeutic groups) the highest reduction in pharmaceutical consumption was not coincidental, because the Spanish Government approved the resolution [47] specifying the list of medicines that the RDL 16/2012 excluded from public provision. This resolution came into force on September 1^st^, 2012.

Most of the therapeutic groups that contained a significant percentage of the excluded drugs (i.e., anti-hyperlipidemics; cardiovascular agents; dermatological; endocrine/metabolic agents; eye, ear, nose and throat preparations; pulmonary drugs) showed notably reduced consumption in the monthly follow-up analysis. In fact, those therapeutic groups that contained some of the excluded drugs slightly recovered their consumption levels when the regression model was run without these drugs. Therefore, and in line with another study [13], the apparent lack of recovery in drug consumption in the medium-term might be the result of the excluded drugs more than the result of the cost-sharing increase. In agreement with other papers [12,13,14,17], the negative impact of the cost-sharing change (without considering in the analysis the list of excluded medicines) seems to be temporary. Despite the findings from the comparison of the analysis with and without the excluded medicines being of interest, we did not detect a total recovery in the consumption when we ran the regression model without the excluded medicines (except for endocrine/metabolic agents and pulmonary drugs (cf. S4 Table)). Consequently, there is a risk of a small loss of medication adherence, as a result of an increase in co-payment percentage, especially, among the most inelastic therapeutic groups that are related to chronic diseases (cardiovascular, anti-hyperlipidemic and dermatological drugs) [16].

In addition, it was no coincidence that there was a slight recovery observed in consumption between October and November 2012, as from July 1^st^ until November 1^st^, 2012, pensioners had to pay provisionally the full 10% of the drug price, with no monthly contribution ceiling. The computer system whereby pensioners could benefit from the monthly ceilings was not available until such date in the Canary Islands [33]. Notwithstanding the above, this highlights that the consumption recovery observed in November was smaller than would have been expected.

These results demonstrate the need to set up co-payments according to the nature of the disease to be treated because, as the existing evidence confirms, the establishment of drug co-payments could deteriorate the health status of low-income people and chronic patients [7]. In this regard, we have to highlight that the current Spanish co-payment system takes into account the income factor but does not sufficiently consider the nature of the disease being treated. Indeed, with the 2012 reform, the list of ATC drugs with reduced contribution, which mainly include medicines for the treatment of chronic diseases, increased the contribution ceiling per pack (cf. Table 1).

Additionally, our analysis of comorbidities (approached as those people who have been dispensed medicines from two or more therapeutic groups), reflected that low-income pensioners strongly decrease their pharmaceutical consumption if we compare them with those who have been dispensed medicines from one therapeutic group. Despite observing a prior increase in their pharmaceutical consumption, probably, with the aim of anticipating drug co-payments changes before the implementation of the reform, the subsequent decrease was much bigger. This circumstance generates a severe risk of loss of medication adherence, probably because patients with comorbidities have a greater pharmaceutical private spending than the rest of patients [11]. Consequently, these low-income pensioners predictably exceeded the monthly contribution ceiling of €8 earlier than those with one disease to treat and this would discourage their adherence. The monthly analysis also confirmed the risk of loss of medication adherence among people with comorbidities. In this regard, patients with comorbidities are mainly patients with several chronic diseases [48] and the establishment of co-payments could deteriorate their health status [7]. Therefore, the increase of the contribution ceiling for ATC drugs with reduced contribution could affect patient with comorbidities in terms of health.

### 4.1. Strengths and limitations of the study

One of the most relevant strengths of the study was the high quality and uniqueness of the dataset. Also, this dataset provided highly individualised information that allowed us to develop a robust study of an individual’s behaviour before and after a co-payment reform, controlling by therapeutic group. Furthermore, our analysis overcomes the limitation of previous studies considering simultaneously three key factors (stockpiling, exclusion of 426 drugs from public provision and date of monthly contribution ceilings implementation) that together with the co-payment increase impacted on consumption after the reform.

A potential limitation of this study is that the specific disease of each individual in the sample was unknown. We only had information about dispensed drugs and, consequently, we were not able to exactly detect comorbidities. However, we explained in the methodology section that CNM and ATC codes allowed us to create the therapeutic groups. Each one of these therapeutic groups has an associated price-elasticity value that served as an orientation guide to the chronicity of the disease to be treated. This is because the price-elasticity for non-chronic is greater (around -0.20) than the price-elasticity for chronic diseases (around -0.08) among the elderly population [4].

## 5. Conclusions

Our first finding is that the negative impact of the cost-sharing change on consumption among low-income pensioners is small. The results also appear to confirm that co-payments do not affect the consumption of middle-income working population. Secondly, the analysis reflects the existence of heterogeneity in the impact of the cost-sharing by therapeutic groups. In this regard, the analysis of stockpiling, a key factor in fully understanding the impact of the reform on drug consumption, revealed that the reduction in consumption was a consequence of prior stockpiling, especially, among the most inelastic therapeutic groups (related to chronic diseases). Nonetheless, the monthly follow-up analysis expanded on this information and showed that the non-recovery observed in any of the therapeutic groups in the subsequent months after the reform, might imply a risk of loss of medication adherence. We especially detected a likely risk among those individuals who were dispensed medicines from two or more therapeutic groups, which are associated with people with comorbidities. Thirdly, the exclusion of 426 drugs from public provision impacted on consumption more than the cost-sharing increase. In any case, as we did not detect a total recovery of consumption when we ran the analysis without the excluded medicines, the risk of a loss of medication adherence was present. This risk was especially marked among the most inelastic therapeutic groups (cardiovascular and anti-hypelipidemics), which are strongly associated with chronic diseases.

Finally, our analysis provides evidence of the existence of heterogeneity in the reform’s impact by therapeutic groups with a higher sensitive to co-payments among chronic patients and individuals with comorbidities than among non-chronic patients. Accordingly, the Spanish Government should consider setting up a co-payment scheme that controls both the nature of the disease being treated (e.g., applying co-payments according to the therapeutic group that each drug belongs to) and the existence of individuals with comorbidities.

## Supporting information

S1 Table Monthly follow-up effect of the cost-sharing change on the pharmaceutical consumption by therapeutic groups (low-income pensioners). The table contains Difference-in-Differences estimates from linear regression models with robust standard errors. Each cell contains results of the model from different therapeutic groups. All regressions include age and age2, and time dummies. Within each cell, we first report the estimated coefficients; we then report in parentheses robust standard errors. The therapeutic groups are sorted by price-elasticity (on the left the most inelastic, while on the right the most elastic).
(DOCX)

S2 Table Monthly follow-up effect of the cost-sharing change on the pharmaceutical consumption by therapeutic groups (middle-income working population). The table contains Difference-in-Differences estimates from linear regression models with robust standard errors. Each cell contains results of the model from different therapeutic groups. All regressions include age and age2, and time dummies. Within each cell, we first report the estimated coefficients; we then report in parentheses robust standard errors. The therapeutic groups are sorted by price-elasticity (on the left the most inelastic, while on the right the most elastic).
(DOCX)

S3 Table Monthly follow-up effect of the cost-sharing on the pharmaceutical consumption by therapeutic groups (without the excluded drugs). The table contains Difference-in-Difference estimates from linear regression models with robust standard errors. Each cell contains results of the model from different therapeutic groups. All regressions include age and age2, and time dummies. Within each cell, we first report the estimated coefficients; we then report in parentheses robust standard errors. This Table only includes the regression models for those therapeutic groups that contain the drugs excluded from coverage.
(DOCX)

S4 Table Monthly follow-up effect of the cost-sharing change on the pharmaceutical consumption by comorbidities. The table contains Difference-in-Differences estimates from linear regression models with robust standard errors. Each cell contains results of the model from different therapeutic groups. All regressions include age and age2, and time dummies. Within each cell, we first report the estimated coefficients; we then report in parentheses robust standard errors.
(DOCX)

S5 Table Monthly prescription percentage of the excluded drugs from coverage, since September 1^st^, 2012. This table only shows those therapeutic groups that contain some medicines included in the list of drugs that changed from a cost-sharing scheme to a payment of 100%, from September 1st, 2012. That is to say, diabetes drugs, gastrointestinal drugs, analgesics/anti-inflammatories, anti-infective and genitourinary groups do not include any drug in the database from the list of excluded medication from the cost-sharing scheme.
(DOCX)

S1 Dataset ZIP file that includes the database text file and an excel file that serves as guide for correctly understand the database variables. (ZIP)

S2 Dataset Zip file that includes an excel file with the Daily Defined Dose variable. (ZIP)
