# Supplementary material for: The effect of a change in co-payment on prescription drug demand in a National Health System: The case of 15 drug families by price elasticity of demand
Source: PLoS One. 2019 Mar 27;14(3):e0213403. doi: 10.1371/journal.pone.0213403 (PMC6436719; doi:10.1371/journal.pone.0213403)
Supplement: S1 Table — The table contains Difference-in-Differences estimates from linear regression models with robust standard errors. Each cell contains results of the model from different therapeutic groups. All regressions include age and age2, and time dummies. Within each cell, we first report the estimated coefficients; we then report in parentheses robust standard errors. The therapeutic groups are sorted by price-elasticity (on the left the most inelastic, while on the right the most elastic). (DOCX) [file pone.0213403.s001.docx]

**S1 Table. Monthly follow-up effect of the cost-sharing change on the pharmaceutical consumption by therapeutic groups (low-income pensioners).**

| Low-income pensioners and low-income working population analysis | | | | | | | |
| --- | --- | --- | --- | --- | --- | --- | --- |
|  | **All groups** | **Cardiovasc.** | **Anti-hyperlip.** | **Endocrine/metabolic** | **Central nervous system** | **Diabetes** | **Derma.** |
| Aug-11 | 0.40 (2.51) | -0.05 (0.81) | -0.47 (0.34) | 0.29 (0.51) | 0.22 (0.55) | 0.42 (0.45) | -0.48 (0.31) |
| sept-11 | -11.50*** (2.23) | -3.23***(0.73) | -1.63***(0.32) | -1.68***(0.45) | -2.04***(0.48) | -1.23***(0.40) | -0.34 (0.30) |
| oct-11 | -27.63*** (2.09) | -8.41***(0.67) | -3.38***(0.29) | -3.85***(0.43) | -3.22***(0.46) | -2.21***(0.38) | -0.64**(0.29) |
| nov-11 | -20.95*** (1.98) | -4.88***(0.63) | -2.12***(0.27) | -2.64***(0.42) | -2.73***(0.43) | -1.64***(0.36) | -0.83***(0.28) |
| Dec-11 | -11.12*** (2.05) | -2.93***(0.64) | -1.41***(0.27) | -1.06***(0.43) | -1.35***(0.45) | -1.04***(0.37) | -0.78***(0.29) |
| Jan-12 | 0.11 (1.98) | -0.69 (0.63) | -0.67***(0.26) | 0.04 (0.42) | 0.08 (0.44) | -0.31(0.37) | -0.09 (0.30) |
| feb-12 | -6.36*** (2.00) | -2.54***(0.63) | -1.31***(0.27) | -0.77 (0.42) | -0.86**(0.45) | -0.85**(0.36) | -0.71***(0.29) |
| mar-12 | 4.96 (2.00) | 0.03 (0.63) | -0.35(0.26) | 1.39***(0.44) | 1.05**(0.46) | -0.26 (0.36) | 0.04 (0.30) |
| Apr-12 | 3.39 (1.96) | -0.86 (0.63) | -0.52**(0.27) | 0.78(0.43) | 0.53 (0.45) | -0.50 (0.36) | -0.24 (0.28) |
| may-12 | 6.82*** (1.98) | 0.13 (0.63) | -0.13(0.26) | 1.32***(0.43) | 1.22***(0.46) | 0.19 (0.37) | -0.21 (0.30) |
| jun-12 | 12.65*** (2.00) | 1.17***(0.64) | 0.15(0.28) | 2.26***(0.46) | 1.95***(0.46) | 0.13 (0.37) | 0.18 (0.30) |
| jul-12 | -16.88*** (2.00) | -4.03***(0.66) | -1.94***(0.28) | -1.47***(0.43) | -0.85 (0.46) | -1.63***(0.35) | -0.95***(0.29) |
| Aug-12 | -7.01*** (2.07) | -1.62***(0.68) | -1.09***(0.30) | 0.16 (0.44) | -0.04 (0.48) | -0.90***(0.37) | -0.38 (0.32) |
| sept-12 | -35.74*** (1.99) | -8.00***(0.66) | -2.40***(0.28) | -3.95***(0.44) | -2.09***(0.46) | -1.14***(0.38) | -1.64***(0.30) |
| oct-12 | -22.83*** (2.03) | -4.73***(0.67) | -0.95***(0.29) | -1.79***(0.45) | -0.02 (0.48) | -1.12***(0.37) | -1.46***(0.30) |
| nov-12 | -22.13*** (2.03) | -5.24***(0.66) | -1.26***(0.29) | -2.43***(0.44) | 0.37 (0.50) | -0.62 (0.36) | -0.91***(0.30) |
| Dec-12 | -24.81*** (2.03) | -6.09***(0.67) | -1.50***(0.28) | -2.68***(0.45) | -0.15 (0.48) | -0.88**(0.37) | -1.51***(0.30) |
| Jan-13 | -13.81*** (2.01) | -2.98***(0.66) | -0.67**(0.29) | -1.42***(0.44) | 1.30***(0.48) | 0.33 (0.37) | -0.83***(0.30) |
| feb-13 | -26.29*** (2.02) | -6.33***(0.67) | -1.62***(0.29) | -3.35***(0.43) | -0.60 (0.48) | -0.84**(0.37) | -1.12***(0.31) |
| mar-13 | -24.71*** (2.00) | -5.97***(0.67) | -1.52***(0.29) | -2.78***(0.44) | -0.20 (0.48) | -0.96***(0.37) | -1.54***(0.30) |
| Apr-13 | -9.30*** (2.00) | -1.70***(0.67) | -0.31(0.29) | -0.84 (0.45) | 2.33***(0.50) | 0.35 (0.37) | -0.95***(0.31) |
| may-13 | -14.33*** (2.03) | -3.63***(0.68) | -0.64**(0.29) | -1.64***(0.44) | 1.57***(0.49) | -0.17 (0.38) | -0.78***(0.32) |
| jun-13 | -21.07*** (2.03) | -5.15***(0.67) | -0.88***(0.29) | -2.54***(0.44) | 0.52 (0.49) | -0.43 (0.38) | -1.06***(0.31) |
|  | **Gastrointestinal drugs** | | **Analgesics/anti-inflammatories** | **Eye, ear, nose and throat preparations** | **Anti-infective** | **Upper respiratory agents** | **Genitourinary agents** |
| Aug-11 | 0.05 (0.07) | | -0.20 (0.25) | 0.17 (0.22) | 0.12 (0.11) | 0.00 (0.08) | 0.20 (0.15) |
| sept-11 | 0.00 (0.07) | | -0.17 (0.25) | 0.04 (0.20) | 0.10 (0.10) | 0.02 (0.08) | -0.39***(0.14) |
| oct-11 | -0.09 (0.06) | | -1.01***(0.24) | -0.07 (0.20) | -0.13 (0.09) | -0.11 (0.08) | -0.48***(0.13) |
| nov-11 | -0.08 (0.06) | | -0.86***(0.23) | -0.16 (0.20) | -0.20**(0.09) | -0.06 (0.08) | -0.35***(0.13) |
| Dec-11 | -0.02 (0.06) | | -0.58***(0.22) | 0.27 (0.20) | 0.02 (0.09) | 0.05 (0.08) | -0.30**(0.13) |
| Jan-12 | 0.00 (0.06) | | -0.02 (0.23) | 0.34 (0.20) | -0.03 (0.09) | 0.10 (0.09) | -0.16 (0.13) |
| feb-12 | -0.03 (0.06) | | -0.31 (0.23) | 0.39 (0.21) | -0.09 (0.09) | 0.05 (0.09) | -0.21 (0.13) |
| mar-12 | -0.01 (0.06) | | 0.15 (0.23) | 0.45**(0.20) | -0.06 (0.10) | 0.08 (0.09) | -0.15 (0.14) |
| Apr-12 | 0.04 (0.06) | | 0.14 (0.23) | 0.69***(0.20) | 0.08 (0.10) | 0.14 (0.08) | 0.18 (0.13) |
| may-12 | 0.02 (0.06) | | 0.47**(0.24) | 0.74***(0.20) | 0.01 (0.10) | 0.18**(0.09) | -0.07 (0.14) |
| jun-12 | 0.15**(0.07) | | 0.99***(0.24) | 1.38***(0.22) | -0.01 (0.10) | 0.11 (0.09) | 0.08 (0.14) |
| jul-12 | -0.05 (0.07) | | -1.39***(0.23) | -0.05 (0.22) | 0.11 (0.09) | -0.04 (0.08) | -0.41***(0.15) |
| Aug-12 | 0.05 (0.07) | | -1.17***(0.24) | 0.36 (0.24) | 0.11 (0.10) | -0.11 (0.09) | -0.31**(0.15) |
| sept-12 | -0.10 (0.07) | | -2.09***(0.24) | -1.96***(0.21) | -0.01 (0.10) | -0.13 (0.09) | -0.57***(0.20) |
| oct-12 | -0.05 (0.07) | | -1.42***(0.24) | -1.63***(0.22) | 0.22**(0.11) | -0.11 (0.09) | -0.19 (0.16) |
| nov-12 | -0.05 (0.07) | | -1.15***(0.24) | -2.19***(0.21) | 0.16 (0.10) | 0.22**(0.10) | -0.32**(0.15) |
| Dec-12 | -0.06 (0.07) | | -1.34***(0.24) | -2.15***(0.20) | 0.37***(0.10) | -0.01 (0.09) | -0.38***(0.15) |
| Jan-13 | -0.08 (0.07) | | -0.65***(0.25) | -1.96***(0.20) | 0.44***(0.11) | -0.01 (0.10) | -0.01 (0.15) |
| feb-13 | -0.03 (0.07) | | -1.50***(0.24) | -2.08***(0.21) | 0.28***(0,11) | -0.14 (0.09) | -0.55***(0.17) |
| mar-13 | -0.07 (0.07) | | -1.14***(0.24) | -2.23***(0.20) | 0.20**(0.10) | -0.08 (0.09) | -0.58***(0.16) |
| Apr-13 | 0.08 (0.07) | | -0.28 (0.25) | -2.03***(0.20) | 0.28***(0.11) | 0.01 (0.09) | -0.15 (0.16) |
| may-13 | 0.06 (0.07) | | -0.64***(0.24) | -1.97***(0.21) | 0.26***(0.10) | 0.06 (0.10) | -0.36**(0.16) |
| jun-13 | 0.00 (0.07) | | -0.95***(0.24) | -2.11***(0.21) | 0.20**(0.09) | -0.01 (0.09) | -0.54***(0.17) |
|  | **Pulmonary drugs** | |  |  |  |  |  |
| Aug-11 | 0.25(0.28) | |  |  |  |  |  |
| sept-11 | -0.31(0.27) | |  |  |  |  |  |
| oct-11 | -0.49 (0.27) | |  |  |  |  |  |
| nov-11 | 0.13 (0.25) | |  |  |  |  |  |
| Dec-11 | 0.23 (0.26) | |  |  |  |  |  |
| Jan-12 | 1.26***(0.26) | |  |  |  |  |  |
| feb-12 | 1.16***(0.28) | |  |  |  |  |  |
| mar-12 | 1.13***(0.28) | |  |  |  |  |  |
| Apr-12 | 1.43***(0.27) | |  |  |  |  |  |
| may-12 | 1.57***(0.30) | |  |  |  |  |  |
| jun-12 | 1.86***(0.29) | |  |  |  |  |  |
| jul-12 | -0.06 (0.27) | |  |  |  |  |  |
| Aug-12 | -0.25 (0.27) | |  |  |  |  |  |
| sept-12 | -0.94***(0.27) | |  |  |  |  |  |
| oct-12 | -1.05***(0.29) | |  |  |  |  |  |
| nov-12 | 0.03 (0.28) | |  |  |  |  |  |
| Dec-12 | -0.22 (0.28) | |  |  |  |  |  |
| Jan-13 | 0.32 (0.29) | |  |  |  |  |  |
| feb-13 | 0.06 (0.28) | |  |  |  |  |  |
| mar-13 | 0.27 (0.29) | |  |  |  |  |  |
| Apr-13 | 0.92***(0.29) | |  |  |  |  |  |
| may-13 | 0.39 (0.29) | |  |  |  |  |  |
| jun-13 | -0.13 (0.29) | |  |  |  |  |  |

The table contains Difference-in-Differences estimates from linear regression models with robust standard errors. Each cell contains results of the model from different therapeutic groups. All regressions include age and age2, and time dummies. Within each cell, we first report the estimated coefficients; we then report in parentheses robust standard errors. The therapeutic groups are sorted by price-elasticity (on the left the most inelastic, while on the right the most elastic).

Significance levels: ***p < 0.01; **p < 0.05.
