# Supplementary material for: The effect of a change in co-payment on prescription drug demand in a National Health System: The case of 15 drug families by price elasticity of demand
Source: PLoS One. 2019 Mar 27;14(3):e0213403. doi: 10.1371/journal.pone.0213403 (PMC6436719; doi:10.1371/journal.pone.0213403)
Supplement: S3 Table — The table contains Difference-in-Difference estimates from linear regression models with robust standard errors. Each cell contains results of the model from different therapeutic groups. All regressions include age and age2, and time dummies. Within each cell, we first report the estimated coefficients; we then report in parentheses robust standard errors. This Table only includes the regression models for those therapeutic groups that contain the drugs excluded from coverage. (DOCX) [file pone.0213403.s003.docx]

**S3 Table. Monthly follow-up effect of the cost-sharing on the pharmaceutical consumption by therapeutic groups (without the excluded drugs).**

| Low-income pensioners and low-income working population analysis | | | | | | | | | |
| --- | --- | --- | --- | --- | --- | --- | --- | --- | --- |
|  | **All groups** | **Anti-hyperlip.** | **Cardio.** | **C. Nervous** | **Derma.** | | **Endocrine/met** | | **Eye, ear, nose.** |
| jul-12 | -13.32***(1.75) | -1.83***(0.26) | -3.66***(0.63) | -0.78 (0.46) | -0.97***(0.29) | | -1.39***(0.37) | | -0.06 (0.15) |
| Aug-12 | -5.17***(1.80) | -1.03***(0.27) | -1.34**(0.64) | 0.09 (0.47) | -0.42 (0.31) | | -0.18 (0.38) | | 0.06 (0.16) |
| sept-12 | -17.72***(1.74) | -1.87***(0.27) | -4.44***(0.62) | -1.51***(0.45) | -1.39***(0.29) | | -1.73***(0.38) | | -0.34**(0.14) |
| oct-12 | -5.12***(1.78) | -0.54**(0.28) | -1.09 (0.63) | 0.58 (0.47) | -1.19***(0.29) | | 0.18 (0.39) | | -0.15 (0.15) |
| nov-12 | -3.24 (1.79) | -0.84***(0.28) | -1.59***(0.62) | 0.97**(0.49) | -0.64**(0.30) | | 0.03 (0.38) | | -0.05 (0.14) |
| Dec-12 | -5.80***(1.79) | -1.04***(0.27) | -2.43***(0.63) | 0.45 (0.47) | -1.24***(0.29) | | -0.14 (0.39) | | -0.01 (0.14) |
| Jan-13 | 5.19***(1.78) | -0.20 (0.28) | 0.67 (0.63) | 1.90***(0.48) | -0.56 (0.30) | | 1.13***(0.39) | | 0.15 (0.13) |
| feb-13 | -7.30***(1.79) | -1.16***(0.28) | -2.67***(0.63) | 0.00 (0.48) | -0.85***(0.30) | | -0.83**(0.38) | | 0.05 (0.15) |
| mar-13 | -5.67***(1.76) | -1.08***(0.27) | -2.31***(0.64) | 0.40 (0.48) | -1.28***(0.29) | | -0.20 (0.39) | | -0.07 (0.13) |
| Apr-13 | 9.73***(1.77) | 0.17 (0.28) | 1.96***(0.64) | 2.93***(0.49) | -0.68**(0.31) | | 1.71***(0.39) | | 0.08 (0.14) |
| may-13 | 4.64***(1.81) | -0.20 (0.28) | 0.02 (0.64) | 2.17***(0.49) | -0.51 (0.32) | | 0.89**(0.39) | | 0.16 (0.14) |
| jun-13 | -2.14 (1.79) | -0.48 (0.27) | -1.49**(0.63) | 1.12**(0.49) | -0.79***(0.31) | | -0.02 (0.38) | | 0.02 (0.14) |
| Low-income pensioners and low-income working population analysis | | | | | | | | | |
|  | **Pulmonary** | | **Up. Respiratory** | | | | | | |
| jul-12 | 0.00 (0.25) | | -0.04 (0.08) | | | | | | |
| Aug-12 | -0.17 (0.25) | | -0.10 (0.09) | | | | | | |
| sept-12 | -0.32 (0.25) | | -0.12 (0.09) | | | | | | |
| oct-12 | -0.43 (0.27) | | -0.10 (0.09) | | | | | | |
| nov-12 | 0.67***(0.26) | | 0.23**(0.10) | | | | | | |
| Dec-12 | 0.41 (0.26) | | 0.00 (0.09) | | | | | | |
| Jan-13 | 0.96***(0.27) | | 0.00 (0.10) | | | | | | |
| feb-13 | 0.69***(0.27) | | -0.13 (0.09) | | | | | | |
| mar-13 | 0.91***(0.27) | | -0.06 (0.09) | | | | | | |
| Apr-13 | 1.56***(0.28) | | 0.02 (0.09) | | | | | | |
| may-13 | 1.03***(0.28) | | 0.07 (0.10) | | | | | | |
| jun-13 | 0.50 (0.27) | | 0.01 (0.09) | | | | | | |
| Middle-income working population and low-income working population analysis | | | | | | | | | |
|  | **All groups** | **Anti-hyperlip.** | **Cardio.** | **C. Nervous** | | **Derma.** | **Endocrine/met** | **Eye, ear, nose.** | |
| jul-12 | 1.34 (0.70) | 0.11 (0.12) | 0.43 (0.24) | 0.23 (0.21) | | 0.21 (0.17) | -0.05 (0.16) | -0.07 (0.05) | |
| Aug-12 | 1.83**(0.75) | 0.07 (0.13) | 0.40 (0.27) | 0.25 (0.23) | | 0.34**(0.17) | -0.17 (0.16) | 0.00 (0.06) | |
| sept-12 | 0.43 (0.72) | 0.02 (0.12) | 0.25 (0.25) | 0.22 (0.22) | | 0.06 (0.16) | -0.10 (0.16) | -0.06 (0.05) | |
| oct-12 | 0.69 (0.75) | 0.07 (0.13) | 0.24 (0.26) | 0.31 (0.24) | | -0.07 (0.15) | -0.13 (0.16) | 0.00 (0.06) | |
| nov-12 | 1.32 (0.76) | 0.09 (0.13) | 0.39 (0.26) | 0.29 (0.23) | | 0.05 (0.15) | 0.06 (0.17) | 0.04 (0.05) | |
| Dec-12 | 1.28 (0.74) | 0.14 (0.12) | 0.38 (0.26) | 0.19 (0.23) | | 0.13 (0.16) | -0.08 (0.16) | -0.04 (0.04) | |
| Jan-13 | 1.77**(0.77) | 0.11 (0.13) | 0.78***(0.26) | 0.18 (0.24) | | 0.18 (0.16) | 0.02 (0.16) | -0.06 (0.05) | |
| feb-13 | 1.53**(0.75) | 0.19 (0.12) | 0.34 (0.25) | 0.34 (0.24) | | 0.29 (0.17) | 0.05 (0.17) | -0.02 (0.04) | |
| mar-13 | 1.09 (0.77) | 0.14 (0.14) | 0.48 (0.26) | 0.15 (0.23) | | 0.12 (0.17) | 0.19 (0.18) | -0.03 (0.05) | |
| Apr-13 | 1.34 (0.78) | 0.14 (0.13) | 0.54**(0.27) | 0.22 (0.25) | | -0.02 (0.15) | -0.03 (0.17) | -0.05 (0.05) | |
| may-13 | 1.95**(0.80) | 0.25 (0.13) | 0.53**(0.27) | 0.38 (0.26) | | 0.13 (0.16) | 0.01 (0.17) | -0.08 (0.05) | |
| jun-13 | 0.98 (0.78) | 0.11 (0.13) | 0.34 (0.27) | 0.35 (0.24) | | 0.06 (0.16) | -0.05 (0.17) | -0.02 (0.05) | |
| Middle-income working population and low-income working population analysis | | | | | | | | | |
|  | **Pulmonary** | | **Up. Respiratory** | | | | | | |
| jul-12 | -0.06 (0.14) | | 0.04 (0.07) | | | | | | |
| Aug-12 | 0.08 (0.16) | | 0.09 (0.07) | | | | | | |
| sept-12 | -0.11 (0.14) | | 0.05 (0.07) | | | | | | |
| oct-12 | -0.09 (0.17) | | 0.01 (0.08) | | | | | | |
| nov-12 | -0.14 (0.16) | | 0.14 (0.08) | | | | | | |
| Dec-12 | 0.11 (0.17) | | 0.16 (0.09) | | | | | | |
| Jan-13 | -0.01 (0.18) | | 0.08 (0.09) | | | | | | |
| feb-13 | -0.08 (0.17) | | 0.11 (0.09) | | | | | | |
| mar-13 | -0.07 (0.16) | | 0.03 (0.08) | | | | | | |
| Apr-13 | -0.14 (0.16) | | 0.06 (0.09) | | | | | | |
| may-13 | -0.02 (0.17) | | 0.02 (0.09) | | | | | | |
| jun-13 | -0.21 (0.16) | | 0.13 (0.08) | | | | | | |

The table contains Difference-in-Difference estimates from linear regression models with robust standard errors. Each cell contains results of the model from different therapeutic groups. All regressions include age and age2, and time dummies. Within each cell, we first report the estimated coefficients; we then report in parentheses robust standard errors. This Table only includes the regression models for those therapeutic groups that contain the drugs excluded from coverage.

Significance levels: ***p < 0.01; **p < 0.05.
