# Supplementary material for: The effect of a change in co-payment on prescription drug demand in a National Health System: The case of 15 drug families by price elasticity of demand
Source: PLoS One. 2019 Mar 27;14(3):e0213403. doi: 10.1371/journal.pone.0213403 (PMC6436719; doi:10.1371/journal.pone.0213403)
Supplement: S4 Table — The table contains Difference-in-Differences estimates from linear regression models with robust standard errors. Each cell contains results of the model from different therapeutic groups. All regressions include age and age2, and time dummies. Within each cell, we first report the estimated coefficients; we then report in parentheses robust standard errors. (DOCX) [file pone.0213403.s004.docx]

**S4 Table.** **Monthly follow-up effect of the cost-sharing change on the pharmaceutical consumption by comorbidities.**

| People who have been dispensed medicines from two or more therapeutic groups | | | | |
| --- | --- | --- | --- | --- |
|  | **Low-income pensioners (with excluded medicines)** | **Low-income pensioners (without excluded medicines)** | **Middle-income working population (with excluded medicines)** | **Middle-income working population (without excluded medicines)** |
| Aug-11 | 3,99 (3,53) | - | 1,15 (4,15) | - |
| sept-11 | -9,72***(3,25) | - | 0,29 (3,90) | - |
| oct-11 | -24,73***(2.96) | - | -0,61 (3,64) | - |
| nov-11 | -21,29***(2.81) | - | 1,44 (3,30) | - |
| Dec-11 | -16,07***(2,86) | - | -3,14 (3,35) | - |
| Jan-12 | -7,67***(2.79) | - | 2,04 (3,34) | - |
| feb-12 | -15,09***(2.79) | - | -1,87 (3,26) | - |
| mar-12 | -8,76***(2,83) | - | 0,84 (3,36) | - |
| Apr-12 | -12,11***(2,80) | - | -1,17 (3,38) | - |
| may-12 | -7,00***(2.82) | - | -0,57 (3,41) | - |
| jun-12 | -4,05 (2.86) | - | 1,65 (3,56) | - |
| jul-12 | -27,27***(2.87) | -24,24***(2,77) | 0,36 (3,36) | 0,56 (3,40) |
| Aug-12 | -20,42***(2.92) | -18,31***(2,81) | 2,04 (3,47) | 1,69 (3,51) |
| sept-12 | -42,56***(2.89) | -29,95***(2,80) | -5,18 (3,46) | -4,27 (3,47) |
| oct-12 | -34,32***(2.88) | -21,94***(2,79) | -2,93 (3,47) | -1,79 (3,50) |
| nov-12 | -32,76***(2.89) | -18,98***(2,80) | -1,05 (3,43) | -0,37 (3,44) |
| Dec-12 | -35,75***(2.87) | -21,78***(2,78) | 0,50 (3,47) | 1,57 (3,49) |
| Jan-13 | -25,62***(2.86) | -11,69***(2,77) | 0,64 (3,47) | 1,53 (3,48) |
| feb-13 | -34,89***(2.87) | -20,95***(2,79) | -1,81 (3,38) | -0,97 (3,41) |
| mar-13 | -36,26***(2.83) | -22,19***(2,75) | -1,12 (3,51) | 0,07 (3,53) |
| Apr-13 | -25,73***(2.86) | -11,59***(2,77) | -2,73 (3,42) | -1,65 (3,41) |
| may-13 | -28,24***(2.91) | -14,28***(2,82) | -0,53 (3,49) | 0,30 (3,51) |
| jun-13 | -34,11***(2.89) | -20,32***(2,80) | -3,77 (3,54) | -2,80 (3,55) |
| People who have been dispensed medicines from only one therapeutic group | | | | |
| Aug-11 | 0,10 (0,66) | - | -0,15 (0,31) | - |
| sept-11 | -1,32** (0,58) | - | -0,47 (0,30) | - |
| oct-11 | -0,73 (0,64) | - | 0,14 (0,30) | - |
| nov-11 | -1,47*** (0,58) | - | -0,35 (0,29) | - |
| Dec-11 | -1,43** (0,60) | - | -0,15 (0,29) | - |
| Jan-12 | 0,01 (0,65) | - | -0,28 (0,29) | - |
| feb-12 | -0,97 (0,65) | - | -0,24 (0,30) | - |
| mar-12 | 0,68 (0,69) | - | -0,20 (0,30) | - |
| Apr-12 | -0,45 (0,65) | - | -0,11 (0,32) | - |
| may-12 | 0,44 (0,64) | - | -0,28 (0,29) | - |
| jun-12 | 0,59 (0,68) | - | -0,05 (0,29) | - |
| jul-12 | 1,23** (0,64) | 1,53**(0,64) | 0,53 (0,33) | 0,49 (0.32) |
| Aug-12 | 1,93*** (0,70) | 2,10***(0,68) | 0,18 (0,32) | 0,25 (0.32) |
| sept-12 | 2,53*** (0,78) | 2,43***(0,77) | 0,09 (0,32) | 0,09 (0.31) |
| oct-12 | 2,91*** (0,74) | 2,87***(0,74) | -0,33 (0,33) | -0,36 (0.33) |
| nov-12 | 2,52*** (0,70) | 2,38***(0,69) | 0,19 (0,36) | 0,18 (0.36) |
| Dec-12 | 1,38** (0,71) | 1,37**(0,70) | 0,46 (0,34) | 0,44 (0.34) |
| Jan-13 | 2,21*** (0,75) | 2,15***(0,74) | 0,57 (0,34) | 0,57 (0.34) |
| feb-13 | 1,56** (0,70) | 1,51**(0,70) | 0,32 (0,33) | 0,30 (0.33) |
| mar-13 | 1,15 (0,70) | 1,05 (0,69) | 0,33 (0,33) | 0,31 (0.33) |
| Apr-13 | 2,29*** (0,77) | 2,22***(0,76) | 0,34 (0,34) | 0,32 (0.33) |
| may-13 | 1,62** (0,74) | 1,55**(0,73) | 0,39 (0,35) | 0,38 (0.35) |
| jun-13 | 2,40*** (0,74) | 2,32***(0,73) | 0,01 (0,34) | -0,02 (0.33) |

The table contains Difference-in-Differences estimates from linear regression models with robust standard errors. Each cell contains results of the model from different therapeutic groups. All regressions include age and age2, and time dummies. Within each cell, we first report the estimated coefficients; we then report in parentheses robust standard errors. The therapeutic groups are sorted by price-elasticity (on the left the most inelastic, while on the right the most elastic).

NOTE: We were not able to exactly know if the individuals of the sample had comorbidities because we did not have information about the specific diseases of each individual. Therefore, we have approached the case of a patient having comorbidities as one who has been dispensed medicines from two or more therapeutic groups. We compared these results with the effect of a patient who has been dispensed medicines from one therapeutic group.

Significance levels: ***p < 0.01; **p < 0.05.
