# Supplementary material for: The effect of a change in co-payment on prescription drug demand in a National Health System: The case of 15 drug families by price elasticity of demand
Source: PLoS One. 2019 Mar 27;14(3):e0213403. doi: 10.1371/journal.pone.0213403 (PMC6436719; doi:10.1371/journal.pone.0213403)
Supplement: S5 Table — This table only shows those therapeutic groups that contain some medicines included in the list of drugs that changed from a cost-sharing scheme to a payment of 100%, from September 1st, 2012. That is to say, diabetes drugs, gastrointestinal drugs, analgesics/anti-inflammatories, anti-infective and genitourinary groups do not include any drug in the database from the list of excluded medication from the cost-sharing scheme. (DOCX) [file pone.0213403.s005.docx]

**S5 Table. Monthly prescription percentage of the excluded drugs from coverage, since September 1^st^, 2012.**

| Time | Anti-hype. | Cardio. | C. Nervous | Derma. | Endocrine. | Eye, ear. | Pulmonary | Up. resp. |
| --- | --- | --- | --- | --- | --- | --- | --- | --- |
| jul-11 | 4.2 | 10.2 | 3.5 | 10.0 | 11.9 | 49,6 | 18 | 13.5 |
| Aug-11 | 3.7 | 9.9 | 3.6 | 10.1 | 13.3 | 51.0 | 18.0 | 10.1 |
| sept-11 | 3.9 | 11.1 | 3.2 | 8.8 | 13.0 | 55.0 | 19.0 | 7.2 |
| oct-11 | 3.8 | 12.5 | 3.2 | 9.3 | 14.9 | 56.0 | 20.8 | 11.8 |
| nov-11 | 3.8 | 11.0 | 2.8 | 14.3 | 13.2 | 55.8 | 21.9 | 11.2 |
| Dec-11 | 3.8 | 10.2 | 2.9 | 14.0 | 13.1 | 54.8 | 25.4 | 9.3 |
| Jan-12 | 3.5 | 10.0 | 2.9 | 12.5 | 13.4 | 55.2 | 27.3 | 18.0 |
| feb-12 | 3.4 | 9.8 | 2.9 | 10.1 | 13.0 | 54.8 | 29.1 | 15.3 |
| mar-12 | 3.7 | 9.5 | 2.8 | 11.6 | 13.0 | 52.3 | 25.3 | 13.1 |
| abr-12 | 3.8 | 9.4 | 2.7 | 12.8 | 12.8 | 54.0 | 21.5 | 12.1 |
| may-12 | 3.6 | 9.1 | 3.0 | 13.3 | 12.8 | 55.0 | 19.7 | 10.0 |
| jun-12 | 3.5 | 9.8 | 2.9 | 11.9 | 13.1 | 55.1 | 17.3 | 9.3 |
| jul-12 | 3.5 | 9.9 | 2.9 | 11.3 | 11.4 | 51.5 | 18.1 | 9.7 |
| Aug-12 | 3.4 | 9.7 | 2.6 | 11.3 | 12.0 | 53.2 | 17.0 | 9.8 |
| sept-12 | 0.0 | 0.3 | 0.1 | 0.4 | 1.5 | 21.3 | 2.3 | 0.0 |
| oct-12 | 0.7 | 0.0 | 0.0 | 0.0 | 1.9 | 22.5 | 2.9 | 0.0 |
| nov-12 | 0.6 | 0.0 | 0.0 | 0.0 | 0.5 | 0.9 | 0.8 | 0.0 |
| Dec-12 | 0.3 | 0.0 | 0.0 | 0.0 | 0.3 | 0.9 | 1.2 | 0.0 |
| Jan-13 | 0.4 | 0.0 | 0.0 | 0.0 | 0.3 | 2.2 | 1.2 | 0.0 |
| feb-13 | 0.4 | 0.0 | 0.0 | 0.0 | 0.4 | 1.4 | 1.8 | 0.0 |
| mar-13 | 0.3 | 0.0 | 0.0 | 0.0 | 0.2 | 0.3 | 0.1 | 0.0 |
| abr-13 | 0.2 | 0.0 | 0.0 | 0.0 | 0.4 | 1.9 | 0.1 | 0.0 |
| may-13 | 0.4 | 0.0 | 0.0 | 0.0 | 0.5 | 1.1 | 0.1 | 0.0 |
| jun-13 | 0.3 | 0.0 | 0.0 | 0.0 | 0.4 | 0.9 | 0.1 | 0.0 |

This table only shows those therapeutic groups that contain some medicines included in the list of drugs that changed from a cost-sharing scheme to a payment of 100%, from September 1st, 2012. That is to say, diabetes drugs, gastrointestinal drugs, analgesics/anti-inflammatories, anti-infective and genitourinary groups do not include any drug in the database from the list of excluded medication from the cost-sharing scheme.
